# Supplementary material for: A functional crosstalk between the H3K9 methylation writers and their reader HP1 in safeguarding embryonic stem cell identity
Source: Stem Cell Reports. 2023 Sep 12;18(9):1775–92. doi: 10.1016/j.stemcr.2023.08.004 (PMC10545489; doi:10.1016/j.stemcr.2023.08.004)
Supplement: Document S1. Figures S1–S5 and supplemental experimental procedures [file mmc1.pdf]

Stem Cell Reports, Volume *18*

Supplemental Information

A functional crosstalk between the H3K9 methylation writers and their reader HP1 in safeguarding embryonic stem cell identity

Lixia Dong, Huaqi Liao, Linchun Zhao, Jingnan Wang, Congcong Wang, Bowen Wang, Yanqi Sun, Lijun Xu, Yin Xia, Shizhang Ling, Xin Lou, and Jinzhong Qin

Supplementary Materials for

A functional crosstalk between the H3K9 methylation writers and their reader HP1 in safeguarding embryonic stem cell identity

Lixia Dong,[1,6] Huaqi Liao,[1,6] Linchun Zhao,[1,6] Jingnan Wang,[1,6] Congcong Wang,[1] Bowen Wang,[1] Yanqi Sun,[1] Lijun Xu,[1] Yin Xia,[2] Shizhang Ling,[3,*] Xin Lou,[4,*] and Jinzhong Qin[1,5,7,*]

*Correspondence author. Jinzhong Qin, Email: qinjz@nju.edu.cn (J.Q.); Xin Lou, Email: xin.lou@zhejianglab.edu.cn(X.L.); Shizhang Ling, Email:lingsz@hotmail.com (S.L.)

The PDF file includes:

Figures. S1 to S5

Other Supplementary Material for this manuscript includes the following:
Tables S1 to S5

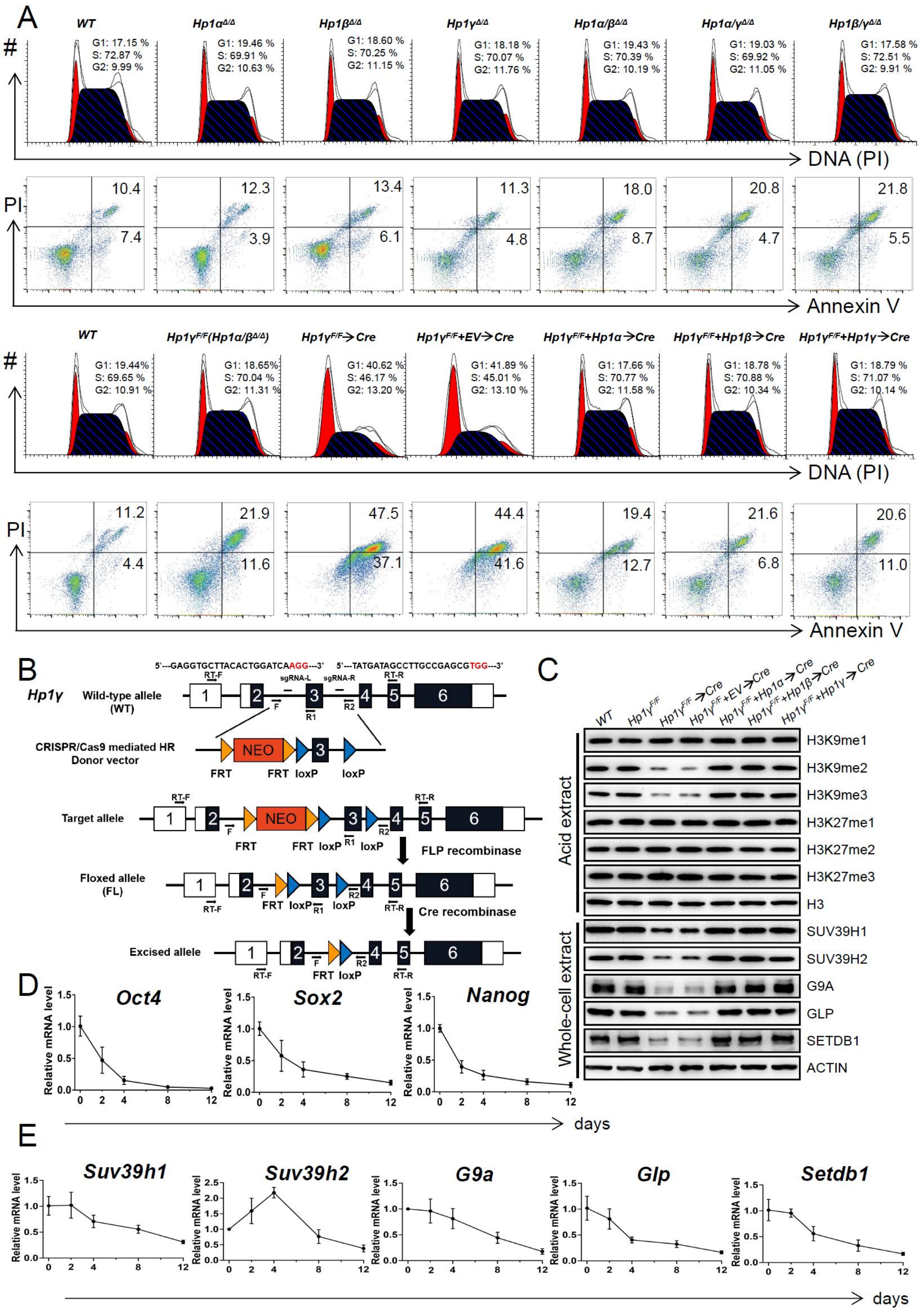
A
WT
G1: 17.15 %
S: 72.87 %
G2: 9.99 %
G1: 19.46 %
S: 69.91 %
G2: 10.63 %
G1: 18.60 %
S: 70.25 %
G2: 11.15 %
G1: 18.18 %
S: 70.07 %
G2: 11.76 %
G1: 19.43 %
S: 70.39 %
G2: 10.19 %
G1: 19.03 %
S: 69.92 %
G2: 11.05 %
G1: 17.58 %
S: 72.51 %
G2: 9.91 %
#
DNA (PI)
PI
Annexin V
10.4
7.4
12.3
3.9
13.4
6.1
11.3
4.8
18.0
8.7
20.8
4.7
21.8
5.5
WT
G1: 19.44%
S: 69.65 %
G2: 10.91 %
G1: 18.65%
S: 70.04 %
G2: 11.31 %
G1: 40.62 %
S: 46.17 %
G2: 13.20 %
G1: 41.89 %
S: 45.01 %
G2: 13.10 %
G1: 17.66 %
S: 70.77 %
G2: 11.58 %
G1: 18.78 %
S: 70.88 %
G2: 10.34 %
G1: 18.79 %
S: 71.07 %
G2: 10.14 %
11.2
4.4
21.9
11.6
47.5
37.1
44.4
41.6
19.4
12.7
21.6
6.8
20.6
11.0
B
5'---GAGGTGCTTACACTGGATCAAGG---3'
5'---TATGATAGCCTTGCCGAGCGTGG---3'
Hp1γ
Wild-type allele (WT)
RT-F
sgRNA-L
sgRNA-R
RT-R
F
R1
R2
CRISPR/Cas9 mediated HR Donor vector
NEO
FRT
loxP
Target allele
FLP recombinase
Floxed allele (FL)
Cre recombinase
Excised allele
C
WT
Acid extract
Whole-cell extract
H3K9me1
H3K9me2
H3K9me3
H3K27me1
H3K27me2
H3K27me3
H3
SUV39H1
SUV39H2
G9A
GLP
SETDB1
ACTIN
D
Oct4
Sox2
Nanog
Relative mRNA level
days
E
Suv39h1
Suv39h2
G9a
Glp
Setdb1
Relative mRNA level
days

Figure S1. The HP1 family is required for maintenance of the pluripotent state in ESCs, related to Figure 1.

(A) Representative cell cycle profiles of propidium iodide–stained ESCs of indicated genotypes obtained by flow cytometric analysis (top). Flow cytometric analysis of apoptosis with annexin V and propidium iodide dual staining (bottom).

(B) Schematic illustration for generation of *Hp1γ* conditional allele via CRISPR/Cas9-mediated gene targeting. The sgRNA sequences were labeled in black and the PAM sequences in red.

(C) Western blot demonstrating the expression levels of selected histone modifications and H3K9 methyltransferases in cells of indicated genotypes. Actin and H3 were taken as loading controls.

(D) and (E) RT-qPCR analysis for (D) *Oct4*, *Sox2*, *Nanog* and (E) H3K9 methyltransferase transcripts in $Hp1\alpha/\beta^{\Delta/\Delta};Hp1\gamma^{F/F}$ ESCs after lenti-*Cre* infection. Relative expression is reflected as fold difference over uninfected ESCs normalized to *β-actin*. Data are expressed as means ± SEM of three biological replicates.

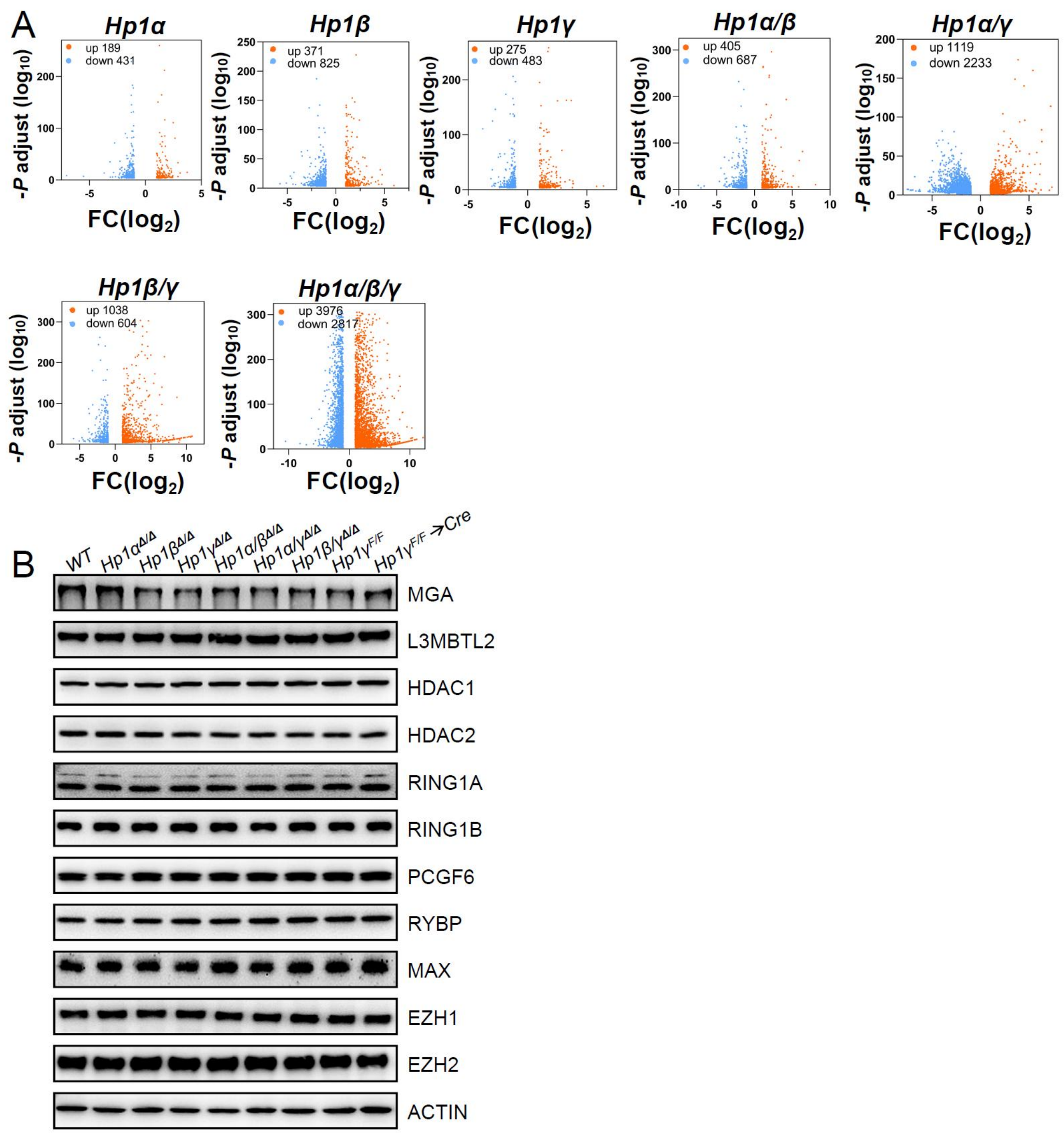

Figure S2. Complete loss of all three *Hp1* paralogs in ESCs exacerbates transcriptional changes in single or double mutants, related to Figure 2.

(A) Volcano plots showing the differentially expressed genes with 2-fold changes in ESCs of indicated genotypes. Blue and red points represent down-regulated represent up-regulated genes, respectively.

(B) Western blot analysis demonstrating expression levels of selected PRC1.6 and PRC2 members in ESCs of indicated genotypes. ACTIN was taken as a loading control.

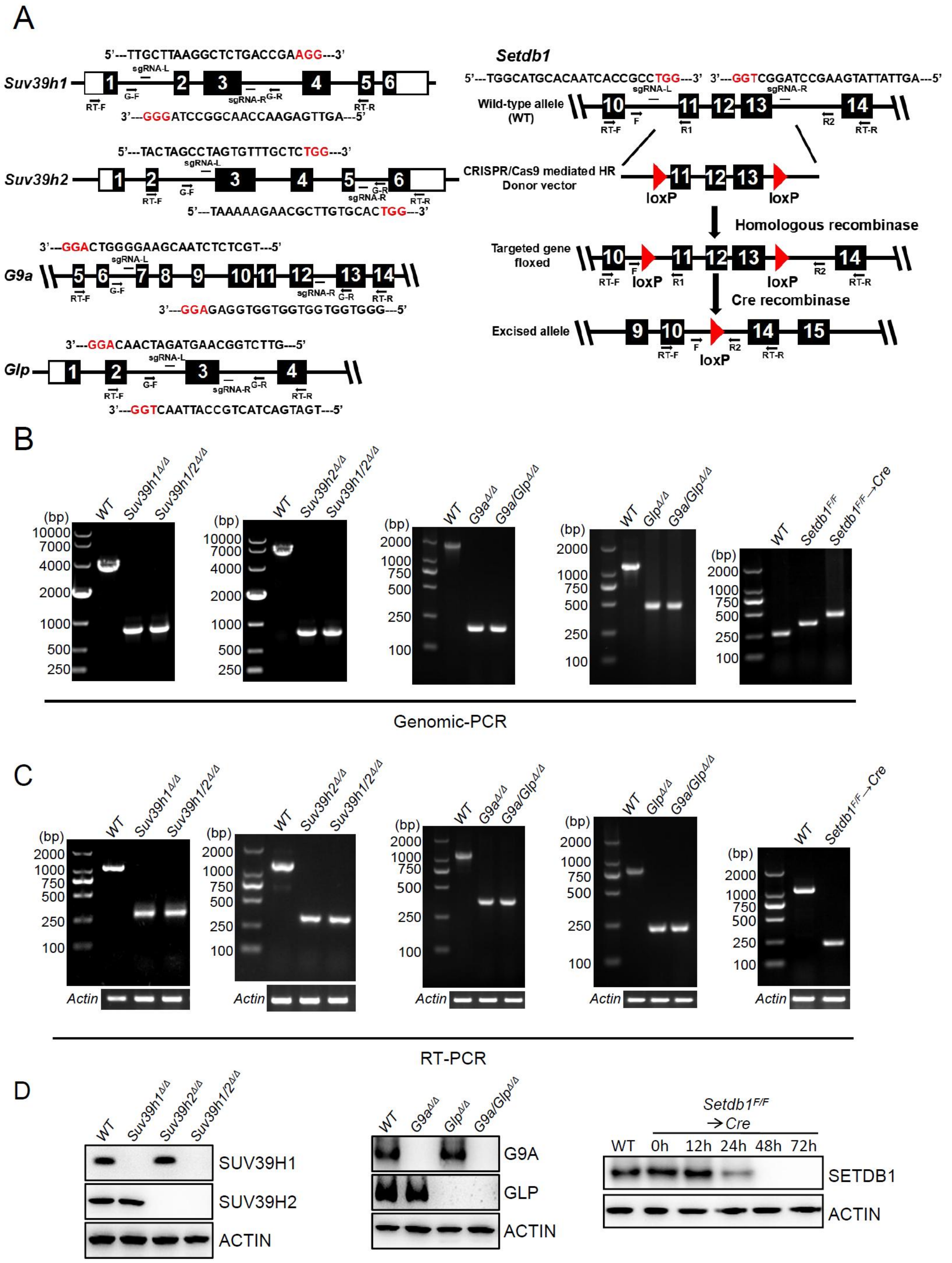

Figure S3. Generation of knockout ESC lines of *Suv39h1*, *Suv39h2*, *G9a*, *Glp* and *Setdb1*, related to Figure 5.

(A) Schematic representation of CRISPR/Cas9 mediated knockout approaches to generate ESCs deficient in *Suv39h1*, *Suv39h2*, *G9a, Glp* and *Setdb1*. SgRNA sequences are shown in black followed by the PAM sequences in red. The locations of genomic PCR primers (G-F, Forward; G-R, Reverse) are shown by arrows.

(B) Genotyping of ESCs with indicated gene deletions using primers located upstream

and downstream of the deleted region.

(C) RT-PCR analysis for residual mRNA revealed a shorter band in the mutants.

(D) Western blot showing the complete ablation of particular gene by using specific antibodiesas indicated. ACTIN was taken as a loading control.

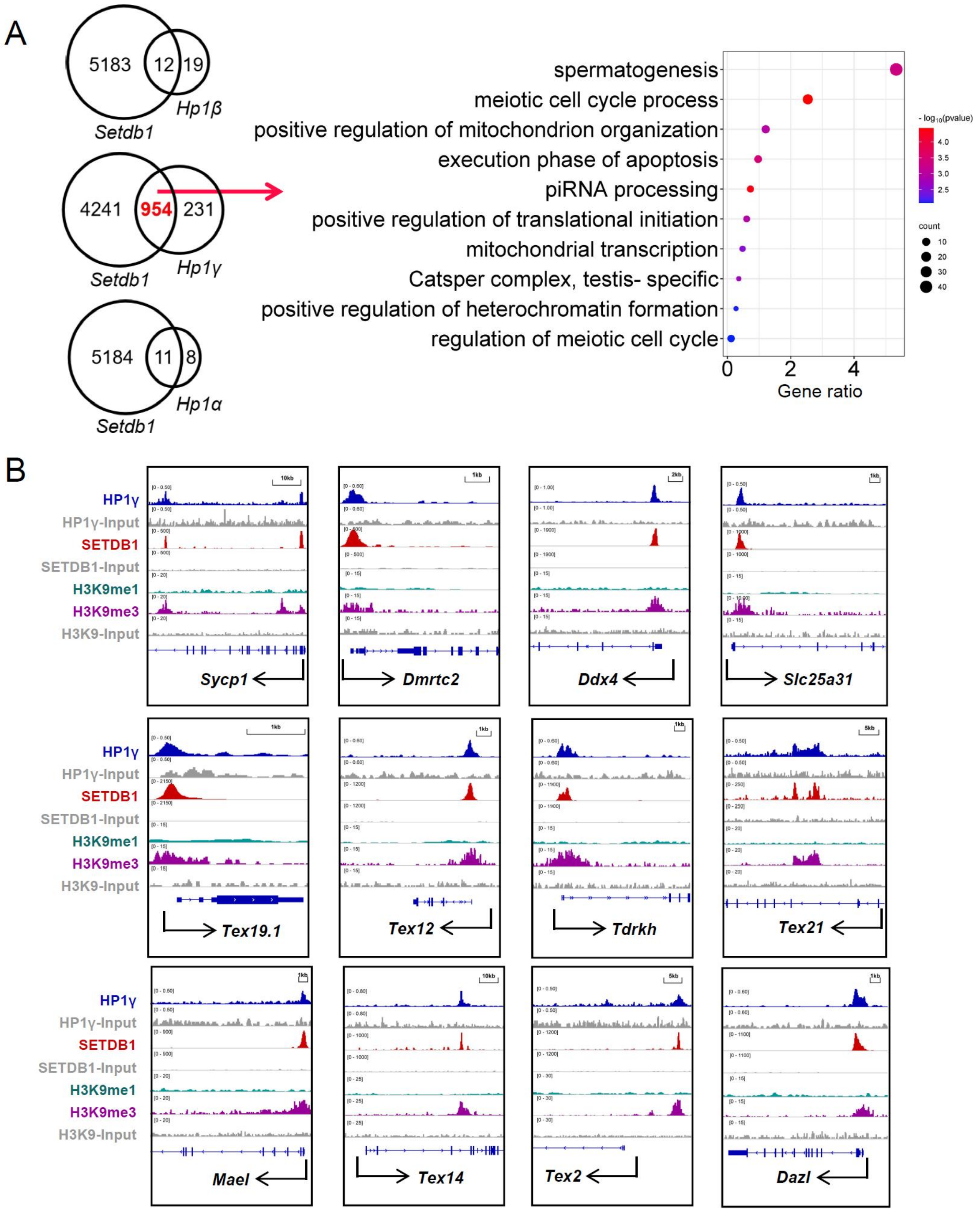

Figure S4. HP1γ and SETDB1 common targets are enriched for germ cell-specific genes in ESCs, related to Figure 6.

(A) Venn diagrams illustrating the overlap of HP1-bound genes and SETDB1-bound genes in ESCs (left). GO analysis of genes co-occupied by HP1γ and SETDB1 (right).

(B) Representative ChIP-seq tracks for HP1γ and SETDB1 at selected target gene loci in wild-type ESCs. Published ChIP-seq data for HP1 and SETDB1 were obtained from NCBI GEO database (accession number GSE94086, GSE71114, GSE108620, GSE126243, GSE54412 and GSE155062).

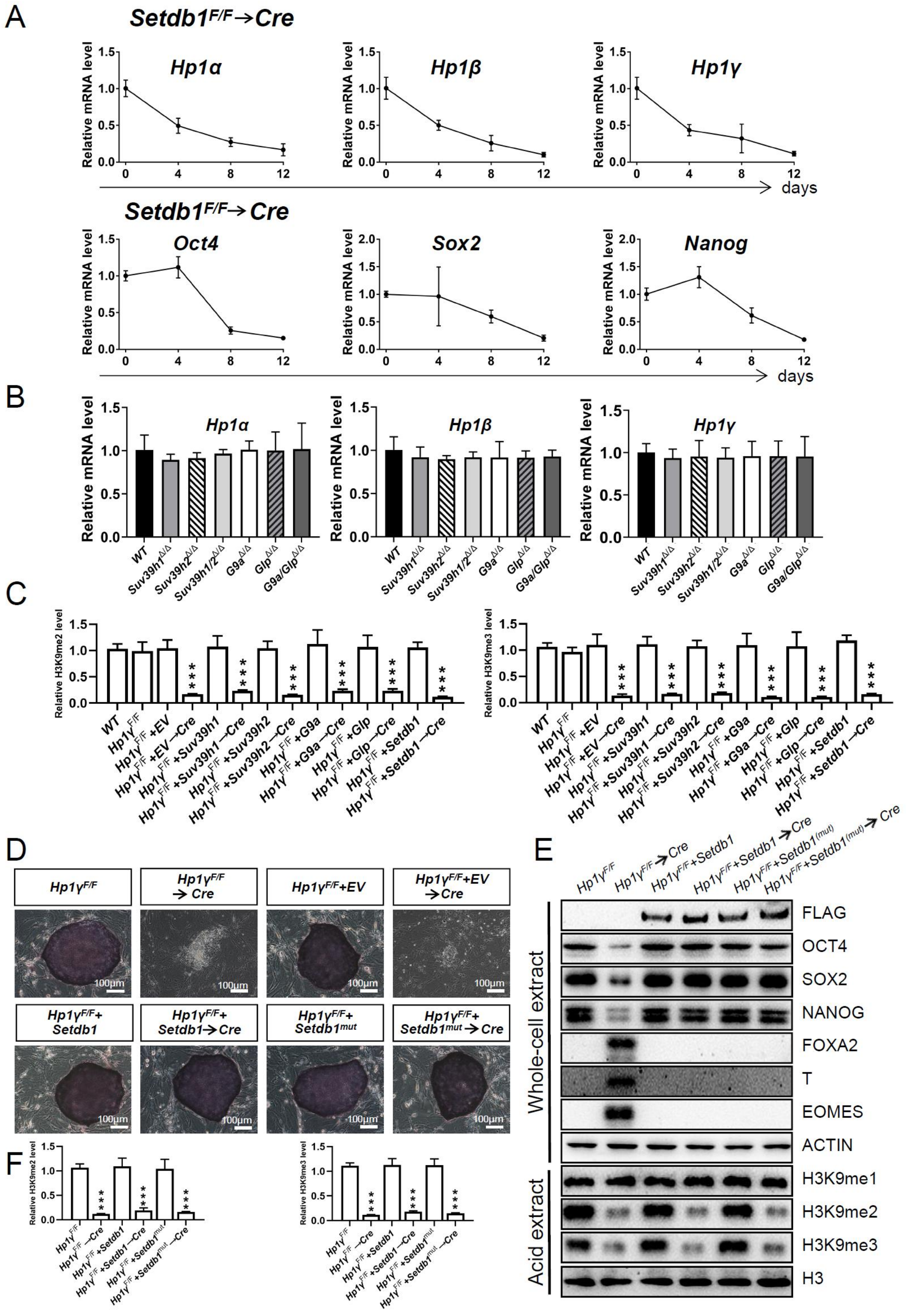

Figure S5. *Setdb1* deficiency results in the dynamic changes in mRNA expression of *Hp1* family genes, related to Figure 6.

(A and B) RT-qPCR analysis for *Hp1*, *Oct4*, *Sox2* and *Nanog* transcripts in *Setdb1*$^{F/F}$ ESCs after lenti-*Cre* infection (A) or in ESCs deficient for *Suv39h1*, *Suv39h2*, *G9a* and *Glp* (B). Relative expression is reflected as fold difference over uninfected or *wildtype* ESCs normalized to *β-actin*. Data are expressed as means ± SEM of three biological replicates. (C) Quantitation of the global levels of H3K9me2 and H3K9me3 in ESCs of indicated genotypes. (D) Representative AP staining images of ESC colonies of indicated genotypes on MEF feeders. Scale bar, 100 μm. *Hp1γ*$^{F/F}$: *Hp1α/β*$^{Δ/Δ}$*;Hp1γ*$^{F/F}$; *EV*: *empty vector*. (E) Western blot demonstrating the expression levels of ectopic *Setdb1*, pluripotency factors, germ layer markers, and histone modifications in cells of indicated genotypes. ACTIN and H3 were taken as the loading controls. *Hp1γ*$^{F/F}$: *Hp1α/β*$^{Δ/Δ}$*;Hp1γ*$^{F/F}$. (F) Quantitation of the levels of H3K9me2 and H3K9me3 in ESCs of indicated genotypes.

Table S1. Genes that are differentially expressed in *Hp1* single knockout vs. control ESCs.

Table S2. Genes that are differentially expressed in *Hp1* double knockout vs. control ESCs.

Table S3. Genes that are differentially expressed in *Hp1* triple knockout vs. control ESCs.

Table S4. Genes that are differentially expressed in *Setdb1*$^{Δ/Δ}$ vs. control ESCs.

Table S5: Key resources used in this study.

Supplemental Experimental Procedures

CRISPR/Cas9-mediated genomic editing

sgRNAs were designed by online CRISPR design tool (http://crispor.tefor.net/). A pair of 20bp-oligonucleotides was annealed and cloned into the BbsI-digested Cas9 and sgRNA-expressing PX459 vector (Addgene plasmid # 62988). sgRNA-expressing plasmids were verified by DNA sequencing. Targeting constructs with appropriate homology arms were generated by standard molecular cloning methods[1]. ESCs were transfected with 1 μg of each Cas9 guide, and 1.5 μg of targeting construct (where appropriate) using Lipofectamine 2000 (ThermoFisher) according to manufacturer's guidelines. The day after transfection, cells were subjected to puromycin (2 μg/ml) selection for 48 hours to eliminate any non-transfected cells. Approximately one week after seeding ESCs on feeder MEFs, individual clones were isolated, expanded, and PCR-screened for the desired genomic modification. All primers are listed in Table S5.

Cloning and plasmid generation

Full-length cDNAs encoding HP1, OCT4, SOX2, NANOG, SUV39H1, SUV39H2, G9A, GLP, and SETDB1 were RT–PCR amplified from mouse mRNA and cloned into lentivirus vector with FLAG epitope (DYKDDDDK) by standard DNA cloning methods. All plasmid

constructions were confirmed by DNA sequencing. Various truncations, deletion, and site mutations were constructed by PCR and confirmed by direct DNA sequencing. Lentiviral production and infection were performed as described previously[2].

Alkaline phosphatase staining

The ESCs were digested with trypsin and then seeded on mitomycin C-treated MEFs for 7-12 days to form colonies, which were washed with phosphate-buffered saline (PBS) twice and fixed with 4% paraformaldehyde for 2 min, and then incubated with staining solution following the protocol recommended by the manufacturer[1]. Finally, the stained colonies were photographed microscopically.

Growth curves

To perform growth curves, ESCs (5×10^4) were seeded in triplicate onto 10 cm MEF-coated dishes in 10 ml of ESC medium on day 0, with culture medium change every other day. Cells were harvested by trypsinization and counted (Trypan blue) every day for 12 days.

Flow cytometry

Cell cycle analysis

The cells were trypsinized, washed twice with ice-cold PBS, and fixed in 70% ethanol in PBS at -20 °C overnight. After fixation, cells were washed with ice-cold PBS and stained with propidium iodide (PI) staining solution, containing 50 μg/mL PI and 200 mg/mL RNase A, at 4°C for 30 min in the dark. Finally, cells were analyzed on a LSRFortessa flow cytometer equipped with Cell Quest software (BD Biosciences) as described previously [2].

Apoptosis analysis

Apoptosis analysis was performed by using Annexin V-FITC/PI apoptosis detection Kit (Yeasen 40302ES60). The cells to be analyzed were collected by trypsin digestion, washed twice with ice-cold PBS, and resuspended in 100 μl binding buffer at room temperature for 10 min in dark which contained 10 μl PI and 5 μl Annexin V-FITC. Fluorescence intensities were analyzed by LSRFortessa flow cytometry as described previously[2].

Western blot analysis

ESCs were lysed with RIPA buffer [50 mM Tris-HCl (pH 8.0), 1% Triton X-100, 0.1% SDS, 150 mM NaCl, 1% sodium deoxycholate, 1 mM EDTA, 10 mg/ml phenylmethylsulfonyl fluoride and Protease Inhibitor Mix (Sigma-Aldrich)] on ice for 30 min. After centrifugation at 13,000 rpm for 15 min at 4°C, the supernatant was collected. The samples were mixed with 5xloading buffer [2% SDS, 125 mM Tris-HCl (pH6.8), 10% glycerol, 1 mg/ml bromophenol blue and 2% β-mercaptoethanol], and heated at 95°C for 10 min. Then, an equal amount of protein (20–50 μg) was separated by with 5–15% SDS-PAGE, blotted to a PVDF membrane (Millipore) and blocked with 5% skim milk powder. The membranes were washed and incubated with the primary antibodies at 4°C overnight. The next day, immunoblotting was performed using the corresponding HRP-conjugated secondary antibody, followed by the visual detection of the protein bands using an enhanced

chemiluminescence detection kit. Antibodies used in this study are listed in Table S5.

Quantitative real-time PCR (RT-qPCR)

Total RNA was extracted by TRIzol reagent (Life Technologies) following the manufacturer's instructions. Complimentary DNA was reverse-transcribed from total RNA using HiScript II 1st Strand cDNA Synthesis Kit (Vazyme) according to the manufacturer's instructions. Samples were analyzed on an ABI 7500 Real Time PCR System (Applied Biosystems) using SYBR green PCR mastermix (Life Technologies) according to the manufacturer's protocol. Results were normalized to *Actin* mRNA levels. Primer sequences are available in Table S5.

RNA sequencing

The total RNA was extracted with TRIzol reagent (Invitrogen), and any contaminating DNA was digested with DNase I. Multiplexed Illumina sequencing libraries were prepared using the TruSeq Stranded Total RNA Library Prep Gold (Illumina, 20020598), and 150 bp paired-end reads were generated with Illumina HiSeq3000 sequencer with a depth of 50 million reads per sample. Differential expression analyses were performed using the R package DESeq2 v1.20 using default parameters. Genes with an absolute log2 fold change of 1 and FDR <0.05 were considered as significant difference. Three biological replicates of each sample were sequenced. The RNA-seq data were deposited at Gene Expression Omnibus under accession number GSE210606.

Analysis of publicly available ChIP-seq data

ChIP-Seq analysis of Publicly available datasets in ESCs were retrieved from the NCBI Gene Expression Omnibus (OCT4 and NANOG:GSE129721, HP1α:GSE94086, HP1β:GSE71114, HP1γ:GSE108620, H3K9me1:GSE54412, H3K9me3:GSE155062 and SETDB1:GSE126243). The ChIP-seq reads were all aligned to the mouse reference genome (GRCm38/mm10) via Bowtie (v2.3.5) in the single-end mode. Then the reads with low quality (MAPQ <20) were removed with the Bam/Sam filter software (version: 1.6). To better visualize the results, alignment files in the BAM format were converted to the read coverage files (BIGWIG format) by the bamCoverage in DeepTools. Subsequently， the converted BIGWIG files were visualized with Integrative Genome Viewer (IGV). And data ranges were set for the appropriate values to underscore differences between each factor level at the target locus. The MACS-determined peak positions were downloaded from ChIP-Atlas (http://chip-atlas.org). Peak overlaps were analyzed using VennDiagram program of Origin (version 2021).

Embryoid body formation

To induce the EB formation, modified hanging drop method from a previously described protocol was followed[2]. Trypsinized cells were suspended in differentiation medium (50 μg/ml Ascorbic acid, 15% fetal bovine serum, 200 μg/ml iron saturated holo-transferrin, 2 mM L-Glutamine, 450 μM monosulfide glycerol, 100 U/ml penicillin-streptomycin, non-essential amino acids and 0.1 mM β-mercaptoethanol) to a concentration of 20,000 cells/ml. 30 μl drops (600 cells) were then placed on the lid of a non-adherent Petri

dish and the lid was upside down. After 72 h incubation, EBs formed at the bottom of the drops were collected and cultured on the rotating shaker at the speed of 60 rpm, with the medium being changed every other day. On day 7 and 12, EBs were collected for morphological imaging and total RNA extraction (Trizol). The genes of interest were analyzed by RT-qPCR. All primers are listed in Table S5.

Teratoma assay

Teratoma formation assay was performed as described previously [1,2]. Briefly, ESCs were MEF depleted and 1 × 10^6 cells were injected subcutaneously into 6-week-old nude mice. After 4 weeks, the teratomas were dissected and fixed in 4% paraformaldehyde for 6 hours at 4℃. Paraffin-embedded tissue was sliced into sections with 5 μm thickness and stained with hematoxylin and eosin.

Supplementary References

1. Qin, J., Wang, C., Zhu, Y., Su, T., Dong, L., Huang, Y., and Hao, K. (2021). Mga safeguards embryonic stem cells from acquiring extraembryonic endoderm fates. Sci Adv *7*. 10.1126/sciadv.abe5689.
2. Qin, J., Whyte, W.A., Anderssen, E., Apostolou, E., Chen, H.H., Akbarian, S., Bronson, R.T., Hochedlinger, K., Ramaswamy, S., Young, R.A., and Hock, H. (2012). The polycomb group protein L3mbtl2 assembles an atypical PRC1-family complex that is essential in pluripotent stem cells and early development. Cell Stem Cell *11*, 319-332. 10.1016/j.stem.2012.06.002.
