# Supplementary material for: A functional crosstalk between the H3K9 methylation writers and their reader HP1 in safeguarding embryonic stem cell identity
Source: Stem Cell Reports. 2023 Sep 12;18(9):1775–92. doi: 10.1016/j.stemcr.2023.08.004 (PMC10545489; doi:10.1016/j.stemcr.2023.08.004)
Supplement: Table S5. Key resources used in this study [file mmc6.docx]

**Table S5**

| REAGENT or RESOURCE | SOURCE | IDENTIFIER |
| --- | --- | --- |
| Antibodies | | |
| Rabbit anti-HP1α | Cell Signaling Technology | Cat# 2616S |
| Rabbit anti-HP1β | Cell Signaling Technology | Cat# 2613S |
| Rabbit anti-HP1γ | Cell Signaling Technology | Cat# 2619S |
| Rabbit anti-Oct4 | Cell Signaling Technology | Cat# 83932S |
| Mouse anti-Sox2 | Cell Signaling Technology | Cat# 4900S |
| Mouse anti-Nanog | Abcam | Cat# ab214549 |
| Mouse anti-Ezh1 | Santa Cruz Biotechnology | Cat# sc-398767 |
| Rabbit anti-Ezh2 | Cell Signaling Technology | Cat# 5246S |
| Rabbit anti-Ring1a | Cell Signaling Technology | Cat# 13069S |
| Rabbit anti-Ring1b | Proteintech | Cat# 16031-1-AP |
| Mouse anti-Hdac1 | Cell Signaling Technology | Cat# 5356S |
| Mouse anti-Hdac2 | Cell Signaling Technology | Cat# 5113S |
| Rabbit anti-Pcgf6 | Abcam | Cat# ab200038 |
| Rabbit anti-Max | Santa Cruz Biotechnology | Cat# sc-197 |
| Mouse anti-Rybp | Santa Cruz Biotechnology | Cat# sc-374235 |
| Rabbit anti-L3mbtl2 | Active Motif | Cat# 39569 |
| Rabbit anti-Mga | Abcam | Cat# ab214814 |
| Rabbit anti-Suv39h1 | Abcam | Cat# ab283262 |
| Rabbit anti-Suv39h2 | Abcam | Cat# ab190870 |
| Mouse anti-G9a | R&D systems | Cat# PP-A8620A-00 |
| Mouse anti-Glp | R&D systems | Cat# PP-B0422-00 |
| Rabbit anti-Setdb1 | Abcam | Cat# ab300573 |
| Mouse anti-Nestin | Cell Signaling Technology | Cat# 33475S |
| Rabbit anti-T | Abcam | Cat# ab209665 |
| Rabbit anti-Foxa2 | Abcam | Cat# ab108422 |
| Rabbit anti-Gata6 | Cell Signaling Technology | Cat# 5851S |
| Rabbit anti-Gata4 | Cell Signaling Technology | Cat# 36966S |
| Rabbit anti-Sox17 | Abcam | Cat# ab224637 |
| Rabbit anti-Eomes | Cell Signaling Technology | Cat# 81493 |
| Rabbit anti-Flag | Sigma-Aldrich | Cat# F1804 |
| Mouse anti-Actin | Proteintech | Cat# 60008-1-Ig |
| Rabbit anti-H3K9me1 | Cell Signaling Technology | Cat# 14186S |
| Rabbit anti-H3K9me2 | Cell Signaling Technology | Cat# 4658S |
| Rabbit anti-H3K9me3 | Cell Signaling Technology | Cat# 13969S |
| Rabbit anti-H3K27me1 | Cell Signaling Technology | Cat# 84932S |
| Rabbit anti-H3K27me2 | Cell Signaling Technology | Cat# 9728S |
| Rabbit anti-H3K27me3 | Cell Signaling Technology | Cat# 9733S |
| Rabbit anti-H2AK119ub1 | Cell Signaling Technology | Cat# 8240S |
| Rabbit anti-H3 | Cell Signaling Technology | Cat# 14269S |
| Chemicals, peptides, and recombinant proteins | | |
| Immobilon Western Chemiluminescent  HRP substrate | Millipore-Sigma | Cat# WBKLS0500 |
| Triton X-100 | Sigma-Aldrich | Cat# Sigma-X-100 |
| Bovine serum albumin (BSA) | Sangon Biotech | Cat# A600332-0100 |
| Ethylenediaminetetracetic acid (EDTA) | Sigma | Cat# E5134 |
| TRIzol | Gibco | Cat# 15596-018 |
| DL2000 DNA Marker | Takara | Cat# 3427A |
| DL10000 DNA Marker | Takara | Cat# 3584A |
| BbsI | NEB | Cat# R0539S |
| T4 DNA Ligase | NEB | Cat# M0202T |
| PrimeSTAR MAX DNA Polymerase | Takara | Cat# R045A |
| CIAP | Takara | Cat# 2250A |
| Gelatin | Sigma | Cat# 48722-500G-F |
| DMSO | Sigma | Cat# D2650 |
| YEAST EXTRACT | OXOID | Cat# LP0021 |
| Fetal bovine serum (FBS) | Gibco | Cat# 10099141 |
| Lipofectamine2000 | Invitrogen | Cat# 11668-019 |
| Hygromycin B | Millipore | Cat# 400052 |
| G418 | Sangon Biotech | Cat# A100859-0001 |
| Critical commercial assays | | |
| HiScriptTM 1st Strand cDNA Synthesis Kit | Vazyme | Cat# R111-02 |
| PowerUp™ SYBR® Green Master Mix | Life | Cat# A25742 |
| Annexin V-FITC/PI | Sangon Biotech | Cat# 40302ES50 |
| Alkaline Phosphatase | Takara | Cat# 2250A |
| Deposited data | | |
| Glp^Δ/Δ^(RNA-Seq) | (Daniela Mayer et al., 2020) | GSE131015 |
| G9a^Δ/Δ^(RNA-Seq) | (Daiki Tatsumi et al., 2018) | GSE102423 |
| shSuv39h1(RNA-Seq) | N/A | GSE99155 |
| shSuv39h2(RNA-Seq) | N/A | GSE99155 |
| HP1α^Δ/Δ^(RNA-Seq) | This article | GSE210606 |
| HP1β^Δ/Δ^(RNA-Seq) | This article | GSE210606 |
| HP1γ^Δ/Δ^(RNA-Seq) | This article | GSE210606 |
| HP1α/β^Δ/Δ^(RNA-Seq) | This article | GSE210606 |
| HP1α/γ^Δ/Δ^(RNA-Seq) | This article | GSE210606 |
| HP1β/γ^Δ/Δ^(RNA-Seq) | This article | GSE210606 |
| HP1α/β/γ^Δ/Δ^(RNA-Seq) | This article | GSE210606 |
| Setdb1^Δ/Δ^(RNA-Seq) | This article | GSE210606 |
| Mga^Δ/Δ^(RNA-Seq) | (Jinzhong Qin et al., 2021) | GSE144141 |
| Pcgf6^Δ/Δ^(RNA-Seq) | (Jinzhong Qin et al., 2021) | GSE144141 |
| L3mbtl2^Δ/Δ^(RNA-Seq) | (Jinzhong Qin et al., 2021) | GSE144141 |
| HP1α(CHIP-Seq) | (Aydan Bulut-Karslioglu et al., 2014) | GSE57092 |
| HP1β(CHIP-Seq) | (Kyoko Hiragami-Hamada et al., 2016) | GSE71114 |
| HP1γ(CHIP-Seq) | (Rupa Sridharan et al., 2013) | GSE44242 |
| Oct4(CHIP-Seq) | (Xiang Sun et al., 2020) | GSE129721 |
| Sox2(CHIP-Seq) | (Xiang Sun et al., 2020) | GSE129721 |
| Nanog(CHIP-Seq) | (Xiang Sun et al., 2020) | GSE129721 |
| Experimental models: Cell lines | | |
| HP1α^Δ/Δ^ | This paper | N/A |
| HP1β^Δ/Δ^ | This paper | N/A |
| HP1γ^Δ/Δ^ | This paper | N/A |
| HP1α/β^Δ/Δ^ | This paper | N/A |
| HP1α/γ^Δ/Δ^ | This paper | N/A |
| HP1β/γ^Δ/Δ^ | This paper | N/A |
| HP1α/β^Δ/Δ^;HP1γ^F/F^ | This paper | N/A |
| Suv39h1^Δ/Δ^ | This paper | N/A |
| Suv39h2^Δ/Δ^ | This paper | N/A |
| Suv39h1/2^Δ/Δ^ | This paper | N/A |
| G9a^Δ/Δ^ | This paper | N/A |
| Glp^Δ/Δ^ | This paper | N/A |
| G9a/Glp^Δ/Δ^ | This paper | N/A |
| Setdb1^F/F^ | This paper | N/A |
| Oligonucleotides | | |
| HP1α upstream sgRNA:  CTCGCCAGTTCTGCCGAGAT | GeneScript | sgRNA |
| HP1α downstream sgRNA:  GGCTGAGTGAGAACTAGTAG | GeneScript | sgRNA |
| HP1β upstream sgRNA:  TACTAGGAAAGTCTAGCGAT | GeneScript | sgRNA |
| HP1β downstream sgRNA:  TCTTGCATAGAAGCCTAGGC | GeneScript | sgRNA |
| HP1γ upstream sgRNA:  GAGGTGCTTACACTGGATCA | GeneScript | sgRNA |
| HP1γ downstream sgRNA:  TATGATAGCCTTGCCGAGCG | GeneScript | sgRNA |
| Setdb1 upstream sgRNA:  TGGCATGCACAATCACCGCC | GeneScript | sgRNA |
| Setdb1 downstream sgRNA:  AGTTATTATGAAGCCTAGGC | GeneScript | sgRNA |
| G9a upstream sgRNA:  TGCTCTCTAACGAAGGGGTC | GeneScript | sgRNA |
| G9a downstream sgRNA:  GGGTGGTGGTGGTGGTGGAG | GeneScript | sgRNA |
| Glp upstream sgRNA:  GTTCTGGCAAGTAGATCAAC | GeneScript | sgRNA |
| Glp downstream sgRNA:  TGATGACTACTGCCATTAAC | GeneScript | sgRNA |
| Suv39h1 upstream sgRNA:  TTGCTTAAGGCTCTGACCGA | GeneScript | sgRNA |
| Suv39h1 downstream sgRNA:  AGTTGAGAACCAACGGCCTA | GeneScript | sgRNA |
| Suv39h2 upstream sgRNA:  TACTAGCCTAGTGTTTGCTC | GeneScript | sgRNA |
| Suv39h2 downstream sgRNA:  TAAAAAGAACGCTTGTGCAC | GeneScript | sgRNA |
| HP1α forward primer:  CACTGCATATGCTAGCGCCT | GeneScript | Genomic-PCR |
| HP1α reverse primer:  TTTGGTTCCCAGCACTCACT | GeneScript | Genomic-PCR |
| HP1β forward primer:  CCCATTTAGGCATCCTGCCA | GeneScript | Genomic-PCR |
| HP1β reverse primer:  ACAAGTCTAAGGCAAACTTGGG | GeneScript | Genomic-PCR |
| HP1γ forward primer:  TCGGTGAAGCAGAACTTTTGT | GeneScript | Genomic-PCR |
| HP1γ reverse primer1:  TCTTCAGGCTCTGCCTCTTC | GeneScript | Genomic-PCR |
| HP1γ reverse primer2:  GTATAGCCCTGGCTGTCCTG | GeneScript | Genomic-PCR |
| Setdb1 forward primer:  TTGCACTCACAGAAATCCAC | GeneScript | Genomic-PCR |
| Setdb1 reverse primer1:  GCTTAAACTGGATTCCAGTA | GeneScript | Genomic-PCR |
| Setdb1 reverse primer2:  CAGATCTTGGTTATGGAAAC | GeneScript | Genomic-PCR |
| G9a forward primer:  CAGGTAGTGGCCAGGACTA | GeneScript | Genomic-PCR |
| G9a reverse primer:  TCTCCATGCCCTCGCATTC | GeneScript | Genomic-PCR |
| Glp forward primer:  GACAGGCATGGGTTTTCTCT | GeneScript | Genomic-PCR |
| Glp reverse primer:  ACTTCACTCAATGCCCAGGA | GeneScript | Genomic-PCR |
| Suv39h1 forward primer:  GCCTTTCCTGGGTCTAAAGG | GeneScript | Genomic-PCR |
| Suv39h1 reverse primer:  CTTTCCCAGGACAACCTGAA | GeneScript | Genomic-PCR |
| Suv39h2 forward primer:  ATGGCATCTGCTGACCTTG | GeneScript | Genomic-PCR |
| Suv39h2 reverse primer:  AGGACAGCCAGGGCTACATA | GeneScript | Genomic-PCR |
| HP1α forward primer:  AGGTGGATCAACAGGCACAC | GeneScript | RT-PCR |
| HP1α reverse primer:  GCCACGTCAGTCTCTCTTCA | GeneScript | RT-PCR |
| HP1β forward primer:  AGTTCTTGATCGGCGAGTTGT | GeneScript | RT-PCR |
| HP1β reverse primer:  GTCAGTAGCTCCAATAATCCGC | GeneScript | RT-PCR |
| HP1γ forward primer:  GGGAGACTCTGCAGGATCC | GeneScript | RT-PCR |
| HP1γ reverse primer:  AAAGCCCCTTGGTTTGTCAG | GeneScript | RT-PCR |
| Setdb1 forward primer:  GATGACAAAAGATGTGAGTG | GeneScript | RT-PCR |
| Setdb1 reverse primer:  GTGGGCAGACACTCTTCTAG | GeneScript | RT-PCR |
| G9a forward primer:  GCTGAACTCTGGTAGCCTG | GeneScript | RT-PCR |
| G9a reverse primer:  GGTGGAATCGCAGCTTCTTC | GeneScript | RT-PCR |
| Glp forward primer:  AGTTCTGGCCAAGCAAGAG | GeneScript | RT-PCR |
| Glp reverse primer:  TGTCTGGGACTTTGTGGTG | GeneScript | RT-PCR |
| Suv39h1 forward primer:  AAGGGGAGGAAGAAGTGGAA | GeneScript | RT-PCR |
| Suv39h1 reverse primer:  CAGATGGTTCTTGTGGCAAA | GeneScript | RT-PCR |
| Suv39h2 forward primer:  CGATTGGAATCACCAAAAGG | GeneScript | RT-PCR |
| Suv39h2 reverse primer:  GCAATGACTTGAGCCATGAA | GeneScript | RT-PCR |
| Nanog forward primer:  ATGCGGACTGTGTTCTCTCA | GeneScript | qPCR |
| Nanog reverse primer:  CCGCTTGCACTTCATCCTTT | GeneScript | qPCR |
| Sox2 forward primer:  CGCGGAGTGGAAACTTTTGT | GeneScript | qPCR |
| Sox2 reverse primer:  CGGGAAGCGTGTACTTATCC | GeneScript | qPCR |
| Oct4 forward primer:  GGATGGCATACTGTGGACCT | GeneScript | qPCR |
| Oct4 reverse primer:  TCTCCAACTTCACGGCATTG | GeneScript | qPCR |
| Sox17 forward primer:  GCCGAGCCAAAGCGG | GeneScript | qPCR |
| Sox17 reverse primer:  GTCAACGCCTTCCAAGACTTG | GeneScript | qPCR |
| Gata6 forward primer:  CCCACTTCTGTGTTCCCAATTG | GeneScript | qPCR |
| Gata6 reverse primer:  TTGGTCACGTGGTACAGGCG | GeneScript | qPCR |
| Gata4 forward primer:  AAACCAGAAAACGGAAGCCC | GeneScript | qPCR |
| Gata4 reverse primer:  ATAGTGAGATGACAGCCCGG | GeneScript | qPCR |
| Foxa2 forward primer:  CCCTACGCCAACATGAACTCG | GeneScript | qPCR |
| Foxa2 reverse primer:  GTTCTGCCGGTAGAAAGGGA | GeneScript | qPCR |
| Nestin forward primer:  AGGTGTCAAGGTCCAGGATG | GeneScript | qPCR |
| Nestin reverse primer:  AAGGAAGCAGACTCAGACCC | GeneScript | qPCR |
| Klf4 forward primer:  GACATCAATGACGTGAGCCC | GeneScript | qPCR |
| Klf4 reverse primer:  TGGGCTTCCTTTGCTAACAC | GeneScript | qPCR |
| Flk1 forward primer:  GCTTGCTCCTTCCTCATCTC | GeneScript | qPCR |
| Flk1 reverse primer:  CCATCAGGAAGCCACAAAGC | GeneScript | qPCR |
| Fgf4 forward primer:  TCTGCCCAACAACTACAACG | GeneScript | qPCR |
| Fgf4 reverse primer:  GAGGGGTAGGGTGTGCTTC | GeneScript | qPCR |
| Fgf5 forward primer:  TTGCGACCCAGGAGCTTAAT | GeneScript | qPCR |
| Fgf5 reverse primer:  CTACGCCTCTTTATTGCAGC | GeneScript | qPCR |
| Krt18 forward primer:  CTGGGGCCACTACTTCAAGA | GeneScript | qPCR |
| Krt18 reverse primer:  ATCTACCACCTTGCGGAGTC | GeneScript | qPCR |
| Tbx2 forward primer:  GCCTGGACAAGAAAGCCAAA | GeneScript | qPCR |
| Tbx2 reverse primer:  TGGTCAGCTTCAGTTTGTGG | GeneScript | qPCR |
| Bmp2 forward primer:  CTGGGGCCACTACTTCAAGA | GeneScript | qPCR |
| Bmp2 reverse primer:  ATCTACCACCTTGCGGAGTC | GeneScript | qPCR |
| Gbx2 forward primer:  TCCCGGCCATTTCGTCTAC | GeneScript | qPCR |
| Gbx2 reverse primer:  CTGGGGATCTGGTGGTGAG | GeneScript | qPCR |
| Ets2 forward primer:  GGCACCAAACTACCCCAAAG | GeneScript | qPCR |
| Ets2 reverse primer:  GTCGTGGTCCTTGGGTTTTC | GeneScript | qPCR |
| Cyp11a1 forward primer:  CGAGTTCACAGGCTGCATAC | GeneScript | qPCR |
| Cyp11a1 reverse primer:  ACTCCGCTAACCACACAGAA | GeneScript | qPCR |
| Msx2 forward primer:  CCTATCAACTCACCCCTGCA | GeneScript | qPCR |
| Msx2 reverse primer:  CATTCAGGAGCAGAGTTGGC | GeneScript | qPCR |
| Bmp4 forward primer:  TCTTTACCGGCTCCAGTCTG | GeneScript | qPCR |
| Bmp4 reverse primer:  AACTCCTCACAGTGTTGGCT | GeneScript | qPCR |
| Hnf1b forward primer:  CTTGGAGGAGTTACTGCCGT | GeneScript | qPCR |
| Hnf1b reverse primer:  CGTGGCCATTGGTGAGAGTA | GeneScript | qPCR |
| Pax6 forward primer:  TCTGCAGGTATCCAACGGTT | GeneScript | qPCR |
| Pax6 reverse primer:  GCAAAGATGGAAGGGCACTC | GeneScript | qPCR |
| Pax9 forward primer:  TATTCTGCGCAACAAGATCG | GeneScript | qPCR |
| Pax9 reverse primer:  GGTGGTGTAGGCACCTTAGC | GeneScript | qPCR |
| Lhx1 forward primer:  CGGAGGCAACTACGACTTCT | GeneScript | qPCR |
| Lhx1 reverse primer:  CTCACTGGAAGGGTGGTGAC | GeneScript | qPCR |
| Dsp forward primer:  TCAGGCTCAATGACAGCATC | GeneScript | qPCR |
| Dsp reverse primer:  TCAGCTTTGAGCCTTTCGAT | GeneScript | qPCR |
| Tcam1 forward primer:  CCTGCTCAGGTCACGTGTTA | GeneScript | qPCR |
| Tcam1 reverse primer:  GGCCCTGTACCTCCAAAGAG | GeneScript | qPCR |
| Tex11 forward primer:  TGTGGTGGCGAAGCATATTA | GeneScript | qPCR |
| Tex11 reverse primer:  AGAAAGCGAAACCCAACAGA | GeneScript | qPCR |
| Tdrkh forward primer:  GCAGCCAATCAGTGTGAGAA | GeneScript | qPCR |
| Tdrkh reverse primer:  ATGGACGTCTCTGGACTGCT | GeneScript | qPCR |
| Ddx4 forward primer:  GGAAGTGGTCGAGGTGGTTA | GeneScript | qPCR |
| Ddx4 reverse primer:  GGAGTCCTCATCCTCTGGTG | GeneScript | qPCR |
| Piwil2 forward primer:  GGAATGCAAAAGCATGAGGT | GeneScript | qPCR |
| Piwil2 reverse primer:  GCTCCAGGATCTTTGTCAGC | GeneScript | qPCR |
| Mael forward primer:  TTCCACGAGGATTTCGATTC | GeneScript | qPCR |
| Mael reverse primer:  TCCATACGCTTCAAACACCA | GeneScript | qPCR |
| Stk31 forward primer:  TCAGGCTGAGTATGGCACTG | GeneScript | qPCR |
| Stk31 reverse primer:  CACAGGGGGATAGGGTTCTT | GeneScript | qPCR |
| Rhox13 forward primer:  GGAGTGCAAGGAGGAAACAA | GeneScript | qPCR |
| Rhox13 reverse primer:  CTCTCCTTGACAGTGGCACA | GeneScript | qPCR |
| Taf7l forward primer:  CAACGTCTCACTGCCTGCTA | GeneScript | qPCR |
| Taf7l reverse primer:  GGTGGAGTAATGCCATGCTT | GeneScript | qPCR |
| Syce1 forward primer:  CCGGATTGAGGTCCTGATTA | GeneScript | qPCR |
| Syce1 reverse primer:  CTTTCCTTCTCCTGGCAGTG | GeneScript | qPCR |
| Tex101 forward primer:  CAATGACTCCCTCTGCAACA | GeneScript | qPCR |
| Tex101 reverse primer:  AGCACTGAGTTGTGCCATTG | GeneScript | qPCR |
| Klf2 forward primer:  CAGGAGCGACAGGTGCAT | GeneScript | qPCR |
| Klf2 reverse primer:  GTCCTCGAAAAGACCGAAGG | GeneScript | qPCR |
| Actin forward primer:  AGCCATGTACGTAGCCATCC | GeneScript | qPCR |
| Actin reverse primer:  CTCTCAGCTGTGGTGGTGAA | GeneScript | qPCR |
| Setdb1 forward primer:  GGCTTATGTTGATCGGCTGT | GeneScript | qPCR |
| Setdb1 reverse primer:  GGAAGCTTCATCCTCAGAGC | GeneScript | qPCR |
| Suv39h1 forward primer:  CCTGCCCTTGGTGTTTCTAA | GeneScript | qPCR |
| Suv39h1 reverse primer:  GCTCCCAGGTGTTTTCTGAG | GeneScript | qPCR |
| Suv39h2 forward primer:  GTGCACGAGGTGCCTTATTC | GeneScript | qPCR |
| Suv39h2 reverse primer:  TGAAACTAGGCAAGGCACAC | GeneScript | qPCR |
| G9a forward primer:  CCACAGAGAGTGTGGATGGA | GeneScript | qPCR |
| G9a reverse primer:  ATGGTCTCCCGCTTAAGGAT | GeneScript | qPCR |
| Glp forward primer:  GGATCTCCGTGTCAGAGGAA | GeneScript | qPCR |
| Glp reverse primer:  CAGAGAGGCACTTCCTGGAG | GeneScript | qPCR |
| Software and algorithms | | |
| IGV | Integrative Genomics Viewer | https://igv.org/ |
| Graphpad Prism | GraphPad | https://www.graphpad.com/ |
| Origin2022 | Origin | https://www.originlab.com/ |
| SnapGene Viewer | SnapGene | https://www.snapgene.com/ |
