# Supplementary material for: A functional crosstalk between the H3K9 methylation writers and their reader HP1 in safeguarding embryonic stem cell identity
Source: Stem Cell Reports. 2023 Sep 12;18(9):1775–92. doi: 10.1016/j.stemcr.2023.08.004 (PMC10545489; doi:10.1016/j.stemcr.2023.08.004)
Supplement: Document S2. Article plus supplemental information [file mmc7.pdf]

Stem Cell Reports
Article
OPEN ACCESS

A functional crosstalk between the H3K9 methylation writers and their reader HP1 in safeguarding embryonic stem cell identity

Lixia Dong,[1,6] Huaqi Liao,[1,6] Linchun Zhao,[1,6] Jingnan Wang,[1,6] Congcong Wang,[1] Bowen Wang,[1] Yanqi Sun,[1] Lijun Xu,[1] Yin Xia,[2] Shizhang Ling,[3,*] Xin Lou,[4,*] and Jinzhong Qin[1,5,*]

[1]State Key Laboratory of Pharmaceutical Biotechnology and MOE Key Laboratory of Model Animals for Disease Study, Model Animal Research Center, Medical School of Nanjing University, Nanjing, China
[2]School of Biomedical Sciences, The Chinese University of Hong Kong, Hong Kong, China
[3]The Translational Research Institute for Neurological Disorders, Department of Neurosurgery, The First Affiliated Hospital (Yijishan Hospital) of Wannan Medical College, Wannan Medical College, Wuhu, China
[4]Research Institute of Intelligent Computing, Zhejiang Lab, Hangzhou 311100, China
[5]Jiangsu Key Laboratory of Molecular Medicine, Medical School of Nanjing University, Nanjing 210093, China
[6]These authors contributed equally
*Correspondence: lingsz@hotmail.com (S.L.), xin.lou@zhejianglab.edu.cn (X.L.), qinjz@nju.edu.cn (J.Q.)
https://doi.org/10.1016/j.stemcr.2023.08.004

SUMMARY

Histone H3 lysine 9 (H3K9) methylation, as a hallmark of heterochromatin, has a central role in cell lineage and fate determination. Although evidence of a cooperation between H3K9 methylation writers and their readers has started to emerge, their actual interplay remains elusive. Here, we show that loss of H3K9 methylation readers, the *Hp1* family, causes reduced expression of H3K9 methyltransferases, and that this subsequently leads to the exit of embryonic stem cells (ESCs) from pluripotency and a reciprocal gain of lineage-specific characteristics. Importantly, the phenotypes of *Hp1*-null ESCs can be rescued by ectopic expression of *Setdb1*, *Nanog*, and *Oct4*. Furthermore, *Setdb1* ablation results in loss of ESC identity, which is accompanied by a reduction in the expression of *Hp1* genes. Together, our data support a model in which the safeguarding of ESC identity involves the cooperation between the H3K9 methylation writers and their readers.

INTRODUCTION

The covalent modification of histone tails is thought to play essential roles in chromatin organization and transcriptional regulation of eukaryotic genes (Strahl and Allis, 2000). Histone H3 lysine 9 (H3K9) methylation, a characteristic mark of gene repression, is closely associated with heterochromatin formation, which is essential for the maintenance of genome stability (Padeken et al., 2022). It is established by members of the family of SET domain-containing histone methyltransferases that target distinct regions of the genome (Rea et al., 2000). SUV39H1, SUV39H2 (also called KMT1A and KMT1B, respectively), and SETDB1 are thought to catalyze H3K9me2 and H3K9me3 (H3K9me2/3), while G9A and GLP (also called EHMT2 and EHMT1, respectively) are the primary enzymes for H3K9me1 and H3K9me2 (H3K 9me1/2) (Padeken et al., 2022; Tachibana et al., 2008).

Heterochromatin protein 1 (HP1) is a conserved nonhistone chromosomal protein that is involved in heterochromatin-mediated gene silencing in many organisms (Canzio et al., 2013; Eissenberg et al., 1990; James and Elgin, 1986; Padeken et al., 2022). Mammalian cells contain three closely related HP1 homologs, HP1α (also known as CBX5), HP1β (CBX1), and HP1γ (CBX3) (James and Elgin, 1986). All HP1 isoforms harbor an N-terminal chromo-domain (CD) and a C-terminal chromo shadow domain (CSD), separated by a flexible hinge region. The CD is responsible for binding to H3K9me2/3 through an aromatic cage (Jacobs and Khorasanizadeh, 2002; Nielsen et al., 2002), while the CSD is required for dimerization, which provides a platform to recruit diverse HP1-binding partners, including transcription co-repressors and H3K9 methyltransferases (Bannister et al., 2001; Canzio et al., 2013; Cowieson et al., 2000; Nozawa et al., 2010). The interaction between HP1 and H3K9 methyltransferases is thought to be important for the spreading and inheritance of heterochromatin. HP1 tethers H3K9 methyltransferases to H3K9me2/3-enriched chromatin through this interaction, thereby promoting further deposition of H3K9me2/3 (Bannister et al., 2001; Richards and Elgin, 2002).

Although it is well established that H3K9 methylation is essential for the establishment and maintenance of the silent chromatin state, evidence for its role in cell fate determination is also emerging (Nicetto et al., 2019; Nicetto and Zaret, 2019; Padeken et al., 2022). H3K9me3 domains have been shown to act as a major obstacle during the conversion of terminally differentiated cells into induced pluripotent stem cells (Chen et al., 2013; Soufi et al., 2012). While single *Suv39h1/2* knockout mice show no developmental defects, double null mice are characterized by perinatal lethality associated with genome instability (Peters et al., 2001). *G9a*- or *Glp*-null mice are early embryonic lethal (E9.5) and exhibit gross morphological abnormalities across all developing cell lineages (Tachibana et al., 2002). *Setdb1*-null mice display early embryonic lethality at the peri-implantation stage and

This is an open access article under the CC BY license (http://creativecommons.org/licenses/by/4.0/).

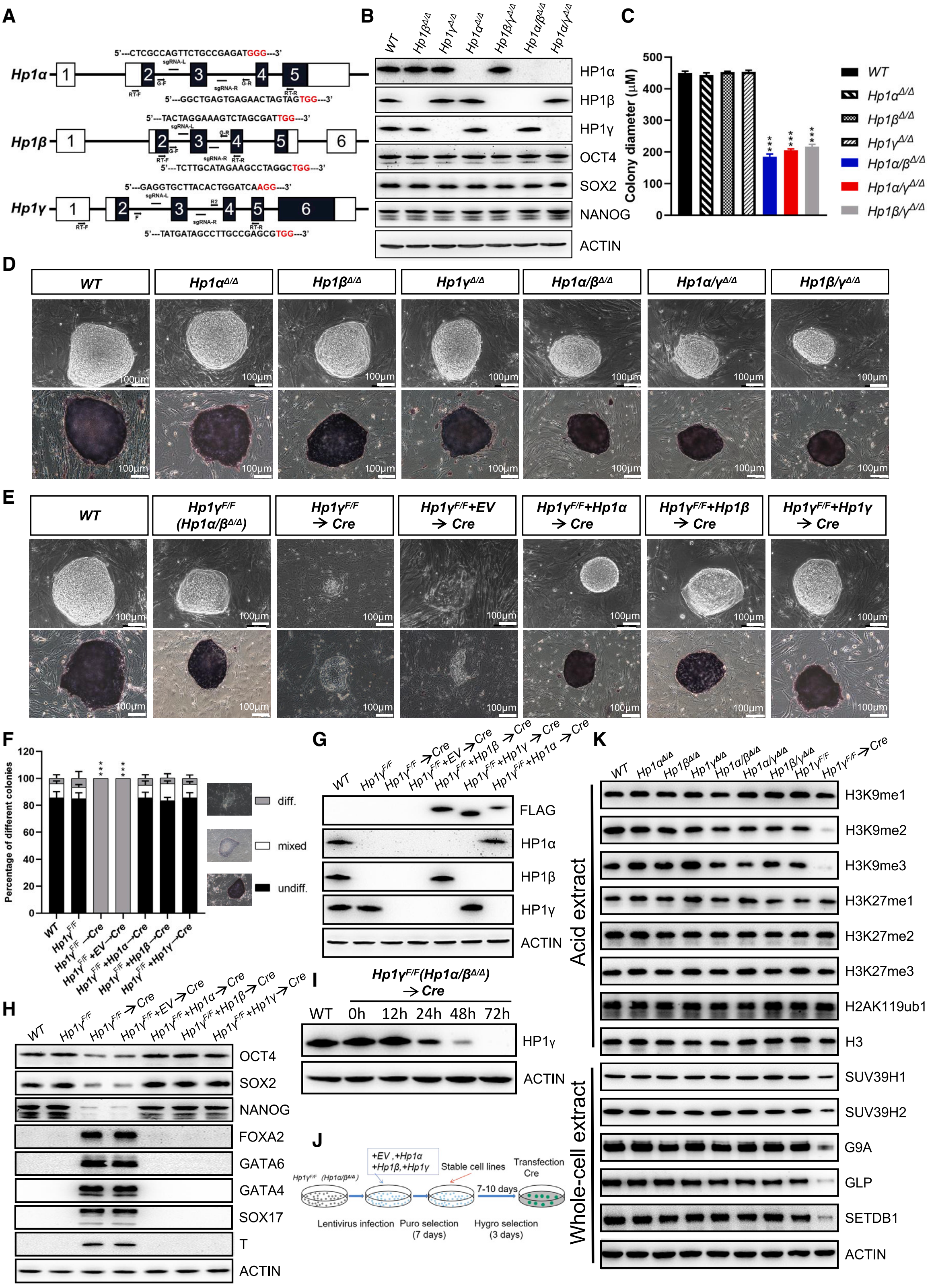

(legend on next page)

embryonic stem cell (ESC) survival *in vitro* is compromised (Dodge et al., 2004). Further studies of *Setdb1* reveal that it contributes to repression of a subset of genes encoding developmental regulators in ESCs (Bilodeau et al., 2009; Yeap et al., 2009; Yuan et al., 2009). *Hp1α*-null mice are viable and fertile and display no obvious morphological abnormalities, whereas *Hp1β*-deficient mice display perinatal lethality and exhibit distorted neurogenesis (Aucott et al., 2008). A dramatic loss of germ cells before meiosis is observed in *Hp1γ*-deficient mice (Abe et al., 2011). The relatively mild phenotypes in single *Hp1* mutants suggest partially redundant functions of the *Hp1* family. Interestingly, a recent work demonstrated that HP1 family members act redundantly to maintain protein stability of H3K9 methyltransferases and to ensure cell viability in ESCs (Maeda and Tachibana, 2022). However, it is unclear whether the reduced expression levels of these enzymes lead to the phenotype observed in *Hp1*-null ESCs.

Here, we use CRISPR/Cas9 technology to generate single or combined *Hp1* mutants and rigorously examine their functions in maintaining self-renewal and pluripotency in ESCs. We show that simultaneous, but not individual, ablation of *Hp1* results in loss of pluripotency in ESCs and triggers compromised self-renewal and spontaneous differentiation, a phenotype strikingly reminiscent of those observed in *Setdb1*-deficient ESCs. In addition, the defects associated with *Hp1*-deficient ESCs can be largely rescued by ectopic expression of *Setdb1*, *Oct4*, and *Nanog*, indicating that *Hp1* safeguards ESC identity through a *Setdb1*-*Oct4*-*Nanog*-dependent mechanism. In these ESCs deficient for all three *Hp1* members, the expression of the H3K9 methyltransferases, particularly *Setdb1*, is reduced markedly. Similarly, *Setdb1* loss of function also causes attenuated *Hp1* expression. Our results reveal that cross-regulation between the H3K9 methylation writer SETDB1 and its reader HP1 is a key component of the transcriptional control of ESC identity.

RESULTS

The *Hp1* family plays essential and redundant roles in the maintenance of ESC pluripotency

To systematically explore the role of *Hp1* family in ESCs, we first established cohorts of ESCs deficient in one or two *Hp1* paralogs in any combination (Figure 1A). The absence of HP1 protein expression was confirmed by western blot analysis (Figure 1B). These mutant cells were monitored for their ability to form colonies after seeding on mitotically inactivated feeder layers made from mouse embryonic fibroblasts (MEFs). The single or double mutants were viable and maintained a typical undifferentiated state in terms of colony morphology along with a high expression level of alkaline phosphatase (AP) and pluripotency markers (OCT4, SOX2, and NANOG), even after long-term culture (Figures 1B and 1D). Notably, the loss of two of the *Hp1* paralogs gave rise to colonies that were significantly smaller in size, whereas the targeted inactivation of single *Hp1s* had no significant effect on overall cell growth (Figures 1C and 1D). Flow cytometry showed that the impaired growth of double mutants was the result of increased apoptosis and not of altered cell cycle (Figure S1A).

To examine the potential functional redundancy among HP1 family in ESCs and to circumvent the lethality or non-physiological responses associated with the deficiency

Figure 1. The *Hp1* family plays essential and redundant roles in the maintenance of the pluripotent state of ESCs

(A) Schematic of CRISPR/Cas9-mediated knockout approaches to generate ESCs deficient in *Hp1* family. PAM sequences are in red following the single guide RNA (sgRNA) sequence highlighted in black.

(B) Western blot analysis showing the changes in the global levels of HP1 proteins and selected pluripotency factors in ESCs of indicated genotypes. ACTIN was taken as a loading control. *WT, wild-type*.

(C) Bar graphs showing the mean largest diameter of 30 random ESC colonies. Data are presented as the means ± SD of three independent experiments. ***p < 0.001 (Student t test) compared with the control.

(D) Representative phase contrast images (top) and AP staining images (bottom) of ESC colonies of indicated genotypes on MEF feeders. All ESC colonies were photographed on day 7 after seeding single-cell suspensions on inactivated MEF feeder layers. Scale bar, 100 μm.

(E) Representative phase contrast images (top) and AP staining images (bottom) of ESC colonies of indicated genotypes on MEF feeders. Scale bar, 100 μm. $Hp1\gamma^{F/F}$, $Hp1\alpha/\beta^{\Delta/\Delta}$;$Hp1\gamma^{F/F}$; *EV, empty vector*.

(F) Quantitation of colony types formed by cell lines shown in (E). AP-stained colonies were scored in three categories: undifferentiated, mixed (partially differentiated) and differentiated. Data are presented as the means ± SD of three independent experiments. ***p < 0.001 (Student t test) compared with the control.

(G and H) Western blot analysis of protein levels of (G) HP1, (H) pluripotency factors, and germ layer markers in ESCs of indicated genotypes. Anti-FLAG (M2) antibody was used to detect the exogenous FLAG-tagged HP1. ACTIN served as a loading control.

(I) Western blot revealing that HP1γ protein was completely ablation after transfection with *Cre* recombinase. ACTIN served as a loading control.

(J) Schematic diagram of the rescue assay.

(K) Western blot demonstrating the changes in the levels of selected histone modifications and H3K9 methyltransferases in ESCs of indicated genotypes. H3 and ACTIN were taken as loading controls. See also Figure S1.

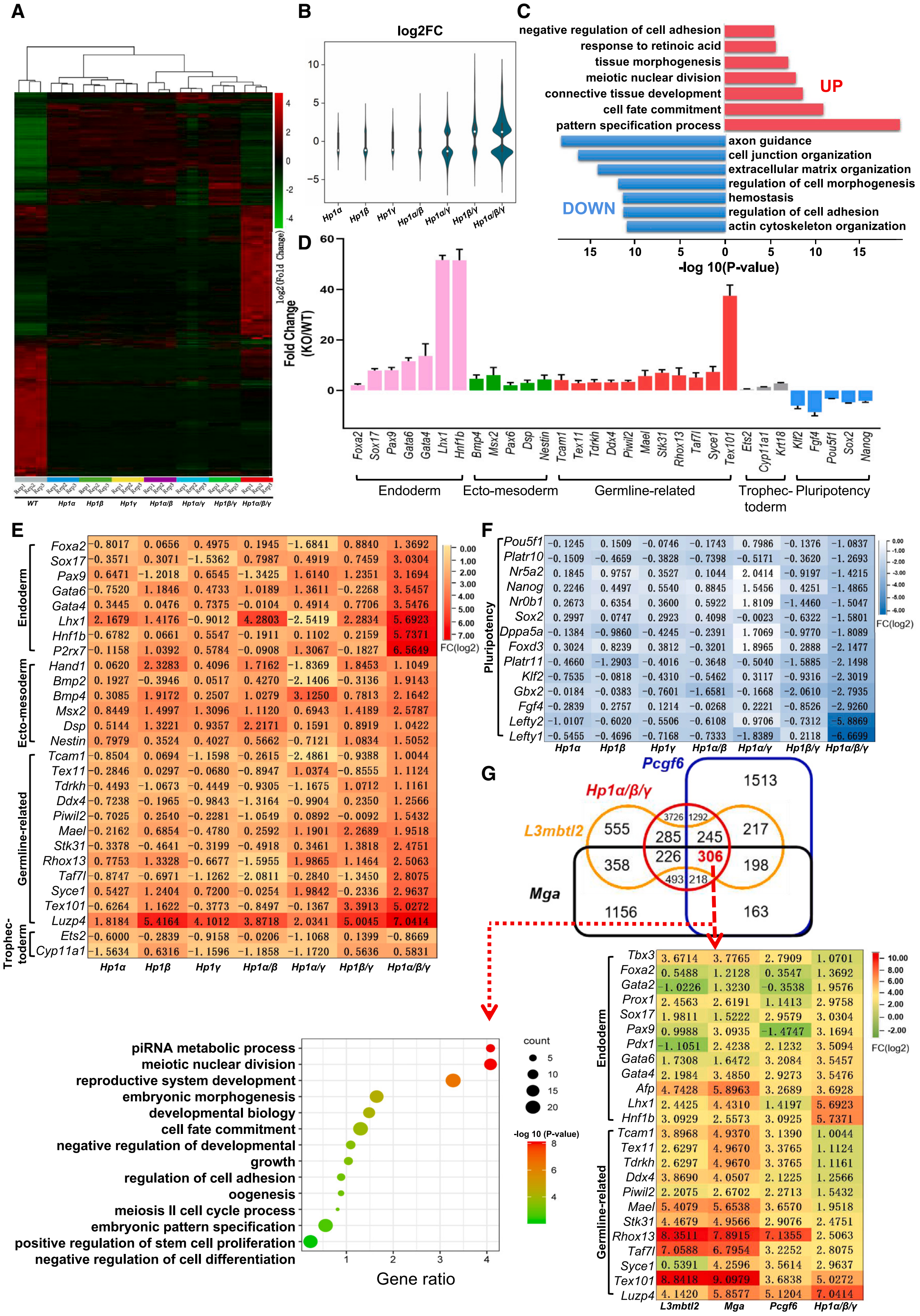

(legend on next page)

for HP1 family, we engineered *Hp1α/β*$^{Δ/Δ}$ ESCs with conditional *Hp1γ*$^{F/F}$ (*Hp1α/β*$^{Δ/Δ}$;*Hp1γ*$^{F/F}$) to eliminate all HP1 activity (Figure S1B). The established *Hp1α/β*$^{Δ/Δ}$;*Hp1γ*$^{F/F}$ lines formed tightly packed colonies with defined borders, stained mostly positive for AP and retained high expression levels of the pluripotency markers (Figures 1E, 1F, and 1H). Transfection of *Cre* recombinase into the established *Hp1α/β*$^{Δ/Δ}$;*Hp1γ*$^{F/F}$ lines led to the efficient ablation of HP1γ proteins (Figure 1I), massive cell death by apoptosis and cell-cycle arrest at G1 phase (Figure S1A), yielding triple knockout (*Hp1α/β/γ*$^{Δ/Δ}$) cells that failed to maintain their pluripotent state, as determined by the reduced expression of pluripotency markers at both the mRNA and protein levels (Figure 1H; Table S3). Therefore, the cells were used for assay after 72 h of transfection throughout this study, unless otherwise stated. Consistent with the observed exit from the pluripotency state, the *Hp1α/β/γ*$^{Δ/Δ}$ cells exhibited differentiated morphology, failed to be stained by AP (Figure 1E), and accompanied by induction of lineage-specific transcriptional regulators, such as FOXA2, GATA4, GATA6, SOX17, and T (Brachyury) (Figure 1H). These results thus uncovered a degree of functional redundancy among HP1 family in preserving ESC pluripotent identity, consistent with data obtained by conditional loss of *Hp1* achieved with a tamoxifen inducible *Cre* (Maeda and Tachibana, 2022). The functional redundancy among members of the HP1 family was independently confirmed by exogenous expression of any *Hp1* paralog, which mostly rescued the detrimental phenotype of *Hp1α/β/γ*$^{Δ/Δ}$ ESCs (Figures 1E–1H, 1J, and S1C).

Interestingly, the markedly decreased levels of global H3K9me2/3, but not H3K9me1, were observed in ESCs with complete deletion of the HP1 family (Figure 1K). In contrast, loss of HP1 did not seem to appreciably affect histone H3 lysine 27 (H3K27) methylation (H3K27me1/2/3). The decrease in H3K9me2/3 levels was reflected by a concomitant decrease in the protein levels of *G9a*, *Glp*, *Setdb1*, and, to a lesser extent, *Suv39h1/2* (Figure 1K). Importantly, in contrast with a rapid decrease in the mRNA levels of *Oct4*, *Sox2*, and *Nanog* after *Cre* transfection, mRNA levels of the H3K9 methyltransferases other than *Glp* remained relatively unchanged or slightly elevated initially (up to 3 days) (Figures S1D and S1E), suggesting a post-transcriptional mechanism of regulation of the expression of H3K9 methyltransferases by HP1 in agreement with data obtained by conditional loss of *Hp1* achieved with a tamoxifen inducible *Cre* (4 days after tamoxifen treatment) (Maeda and Tachibana, 2022). Additionally, mRNA levels of all the H3K9 methyltransferases dramatically decreased 3 or 4 days after *Cre* transfection (Figure S1E). Therefore, the expression of H3K9 methyltransferases in ESCs mediated by HP1 can be achieved both at the transcriptional and post-transcriptional levels. Of note, the level of H3K9me2/3 and H3K9 methyltransferases were maintained in *Hp1α/β/γ*$^{Δ/Δ}$ cells exogenously expressing any one of the *Hp1* paralogs (Figures 1J and S1C), confirming that all HP1 paralogs display the ability to ensure the requisite levels of H3K9 methyltransferases.

HP1 represses expression of lineage-specific genes in ESCs

To gain insight into the molecular mechanisms behind the observed phenotypic changes, we performed RNA sequencing (RNA-seq) analysis in *Hp1* individual and compound mutants (Tables S1, S2, and S3). We observed deregulation of increasing numbers of genes in *Hp1* mutants lacking progressively more *Hp1* paralogs (Figures 2A and 2B). While only 620 (189 up-regulated and 431 down-regulated), 1,196 (371 up-regulated and 825 down-regulated) and 758 (275 up-regulated and 483 down-regulated) genes were deregulated in *Hp1α*, *Hp1β*, and *Hp1γ* single mutants, respectively, when compared with their parental counterpart, cells deleted for

Figure 2. HP1 family acts redundantly to repress expression of lineage-specific genes in ESCs
(A) Heatmap representation of global transcriptional changes in ESCs of indicated genotypes. All these selected genes were significantly differentially expressed in *Hp1* triple knockout cells (log2 FC > 1 or < −1, adjusted $p < 0.05$). For each gene, the *Z* score was calculated based on mean TPM values that were transformed to log2-counts. Hierarchical clustering of all samples was predicated on Euclidean distance metric and agglomerative hierarchical clustering. Up- and down-regulated genes are shown in red and green, respectively. RNA for RNA-seq analysis was obtained from *Hp1α/β*$^{Δ/Δ}$;*Hp1γ*$^{F/F}$ cells transfected with *Cre* or control vector for 72 h in three independent experiments. RNA-seq data for ESCs deficient in one or two *Hp1* paralogs were generated from their constitutive knockout ESCs.
(B) A violin plot comparing log2-fold changes of genes in ESCs of indicated genotypes.
(C) GO analysis of genes up-regulated (top) and down-regulated (bottom) in *Hp1α/β/γ*$^{Δ/Δ}$ cells.
(D) RT-qPCR analysis of germ layer and pluripotency marker expression levels in *Hp1α/β/γ*$^{Δ/Δ}$ cells. All data are normalized to actin and shown relative to control ESCs (set at 1.0). Error bars represent the mean ± SD (n = 3).
(E and F) Heatmaps showing the expression levels of genes involved in (E) germ layer specification and (F) pluripotency maintenance in ESCs of indicated genotypes.
(G) Venn diagram showing intersection between differentially expressed genes in *Hp1α/β/γ*$^{Δ/Δ}$, *Pcgf6*$^{Δ/Δ}$, *L3mbtl2*$^{Δ/Δ}$, and *Mga*$^{Δ/Δ}$ ESCs; $p < 0.05$. Heatmap representation of expression levels of genes involved in endoderm and germ cell specification in ESCs upon *Pcgf6*, *L3mbtl2*, *Mga*, or *Hp1* ablation (bottom). GO analysis of biological functions of differentially overlapped genes with fold changes of more than 2 (left). Published RNA-seq data were acquired from the National Center for Biotechnology Information (NCBI) (GEO: GSE144141). See also Figure S2.

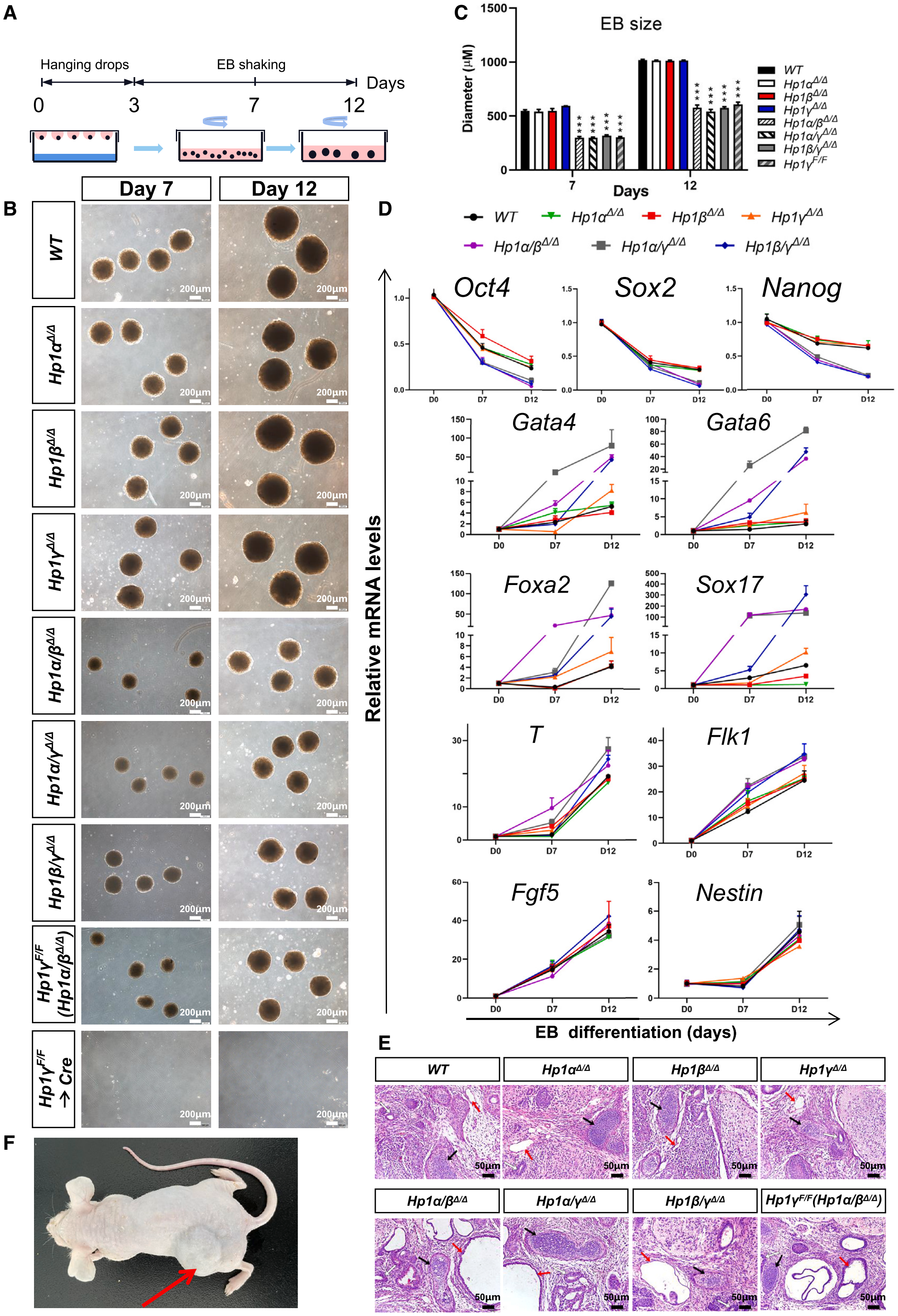

(legend on next page)

two of the *Hp1* paralogs show deregulation of thousands of genes (1,038 up-regulated and 604 down-regulated in *Hp1β/γ*$^{Δ/Δ}$, 1,119 up-regulated and 2,233 down-regulated in *Hp1 α/γ*$^{Δ/Δ}$, and 405 up-regulated and 687 down-regulated in *Hp1α/β*$^{Δ/Δ}$). Complete loss of all three *Hp1* paralogs in ESCs resulted in substantial changes in gene expression (3,976 up-regulated versus 2,817 down-regulated genes) (Figures 2A, 2B, and S2A; Table S3), further supporting the existence of functional redundancy among *Hp1* family. Functional annotation of *Hp1α/β/γ*$^{Δ/Δ}$ up-regulated genes showed enrichment in Gene Ontology (GO) terms related to pattern specification process, cell fate commitment and tissue morphogenesis. Genes down-regulated in *Hp1α/β/γ*$^{Δ/Δ}$ cells were related to regulation of axon guidance and cell junction organization, as well as regulation of cell morphogenesis (Figure 2C).

Importantly, examination of up-regulated genes in *Hp1α/β/γ*$^{Δ/Δ}$ cells also showed a strong over-representation of embryonic germ layer lineage genes, including key endoderm markers (such as *Foxa2*, *Sox17*, *Gata6*, *Gata4*, and *Hnf1b*), markers of ecto-mesoderm (such as *Hand1*, *Bmp4*, *Msx2*, *Pax6*, and *Dsp*). and meiosis- and germline-specific genes (such as *Mael*, *Stk31*, *Taf7l*, *Syce1*, and *Tex101*). In contrast, expression levels of trophectoderm genes (*Ets2* and *Cyp 11a1*), either decreased or remained unaffected in the mutant (Figures 2D–2F; Table S3). Among the genes down-regulated in *Hp1α/β/γ*$^{Δ/Δ}$ cells, our analysis revealed the pluripotency genes, including *Oct4* (*Pou5f1*), *Nr5a2*, *Nanog*, *Sox2*, and *Dppa5a* (Figure 2F), which have been reported to have essential roles in regulating pluripotency in ESCs. Consistent with the results of RNA-seq analysis, by performing RT-qPCR, we confirmed the findings revealed by RNA-seq and demonstrated that while the expression of the germ-layer-specific genes was significantly up-regulated in *Hp1α/β/γ*$^{Δ/Δ}$ cells, their expression was largely unaffected in *Hp1* single or double mutants (Figure 2D; Tables S1, S2, and S3). Together, these results indicate that *Hp1* paralogs act redundantly to silence lineage-specific genes in ESCs.

Of note, a large number of the genes deregulated in *Hp1α/β/γ*$^{Δ/Δ}$ cells were also observed in *L3mbtl2*-, *Mga*-, or *Pcgf6*-deficient ESCs (Figure 2G). Furthermore, among the up-regulated genes, we found that a substantial number involved in germ cell specification and endoderm formation have been previously described to be direct targets of RYBP (Hisada et al., 2012), L3MBT2 (Qin et al., 2012), PCGF6 (Endoh et al., 2017; Zhao et al., 2017), MAX (Maeda et al., 2013), and MGA (Qin et al., 2021), confirming that HP1 associates with members of the PRC1.6 complex (Gao et al., 2012; Ogawa et al., 2002; Qin et al., 2012). However, loss of *Hp1* did not appreciably affect protein levels of the Polycomb group (PcG) family members we examined (Figure S2B). Together, the HP1 family has an essential role in maintaining pluripotency in ESCs.

The HP1 family is essential for proper ESC differentiation

To explore the *in vitro* differentiation potential of *Hp1* mutant ESCs, these cells were examined for their capacity to aggregate in suspension to form embryoid bodies (EBs). To this end, ESCs were induced to differentiate upon the removal of both leukemia inhibitory factor (LIF) and feeder cells and growing in suspension culture (Figure 3A). As shown in Figures 3B and 3C, although all of the *Hp1* single and double mutants remained able to form EBs, *Hp1* double mutant EBs were greatly reduced in size in comparison with single mutants during the differentiation process. We assessed the expression kinetics of marker genes during differentiation. Both *wild-type* and *Hp1* mutant ESCs gradually lost expression of the pluripotency marker *Oct4*, *Nanog*, and *Sox2* after several days of differentiation and showed a similar increase in the embryonic ectoderm markers (*Fgf5* and *Nestin*) (Figure 3D). Strikingly, *Hp1* double mutants showed accelerated down-regulation of *Oct4*, *Nanog*, and *Sox2* after differentiation induction, and expressed significantly increased levels of genes characteristic of extraembryonic endoderm (*Gata4*, *Gata6*, *Foxa2*, and *Sox17*), and to a lesser extent, mesoderm (*T* and *Flk1*).

Figure 3. The HP1 family is essential for proper ES cell differentiation

(A) Schematic illustration of the EB formation.

(B) Phase contrast images of EBs derived from indicated ESCs at day 7 and day 12. Scale bar, 100 mm.

(C) Bar graphs showing the mean largest diameter of 30 random EBs of indicated genotypes. Data are presented as the means ± SD for analysis of three independent experiments. ***$p < 0.001$ (Student t test) compared with the control.

(D) Pluripotency and differentiation gene expression analysis by RT-qPCR of mRNA derived from EBs of indicated genotypes. Error bar represent ± SD (n = 3).

(E) Histological sections of hematoxylin and eosin-stained teratomas of indicated genotypes performed. Of note, teratomas derived from *Hp1* double mutant cells contained structures pertaining to all three germ layers but with an overrepresentation of the endoderm structures (intestinal epithelium). Neural rosette (ectoderm; white arrow), cartilage (mesoderm; black arrow), and gut epithelium (endoderm; red arrow) are shown. Scale bar, 50 μm.

(F) Representative images of mice bearing teratomas 4 weeks after the injection of *wild-type* ESCs (left side, indicated by a red arrow) or *Hp1α/β/γ*$^{Δ/Δ}$ cells (right side). Five mice were injected per condition.

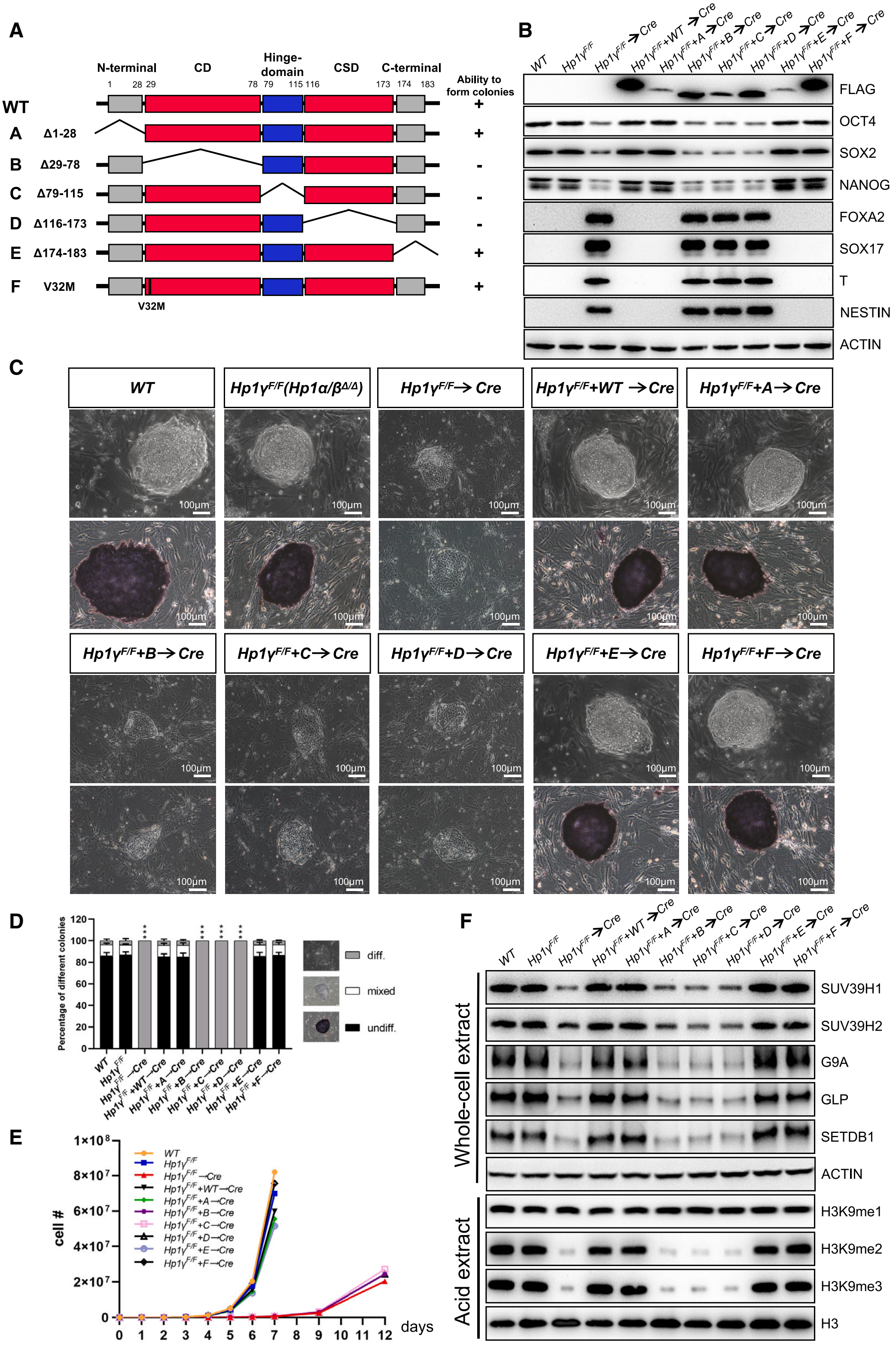

(legend on next page)

To demonstrate the *in vivo* differentiation potential of *Hp1*-mutant ESCs, these cells were injected subcutaneously into immune-deficient mice. Histological analysis revealed that, while *Hp1* single mutants produced teratomas containing all three germ-layer lineages, double mutants developed teratomas containing derivative of all three germ layers, but with an over-representation of the endoderm structures (Figure 3E). Of note, the $Hp1\alpha/\beta/\gamma^{\Delta/\Delta}$ cells failed to generate detectable teratomas upon subcutaneous injection into the nude mice (Figure 3F). These findings demonstrate that *Hp1* family is critical for proper differentiation of ESCs.

The known domains of HP1 are essential for maintaining ESC identity and pluripotent state

As mentioned above, the defects observed in $Hp1\alpha/\beta/\gamma^{\Delta/\Delta}$ cells were mostly restored by re-expression of one *Hp1* family member (Figures 1E–1J), thus providing us with an opportunity to examine HP1 structure and function in ESCs. The HP1 molecule contains an N-terminal chromo domain, a connecting hinge, and a CSD (Figure 4A). Next, we performed a structure-function analysis to define the regions within the HP1 protein that mediate its effects on the maintenance of stem cell identity. The series of FLAG-tagged HP1γ deletion mutants shown in Figure 4A was constructed and introduced into $Hp1\alpha/\beta^{\Delta/\Delta};Hp1\gamma^{F/F}$ ESCs, in which both alleles of the *Hp1α/β* genes were disrupted and the *Hp1γ* gene was floxed and could be conditionally deleted by *Cre* recombinase. Of note, western blot with FLAG-specific antibody confirmed the expression of each truncated protein in ESCs (Figure 4B). To gain insight into the ability of these mutants to rescue the self-renewal defect of $Hp1\alpha/\beta/\gamma^{\Delta/\Delta}$ cells, we examined their capacity to form colonies after culturing on mitotically inactivated MEF feeder layers. As shown in Figures 4C–4E, mutants lacking the first 28 amino acids or the last 10 amino acids fully rescue the colony growth defects in $Hp1\alpha/\beta/\gamma^{\Delta/\Delta}$ cells to the *wild-type* level, while HP1γ mutants in which the chromo, chromo shadow, or hinge domain was deleted abolished its ability to restore the growth defect (Figures 4C–4E). To examine the ability of these mutants to maintain the expression of H3K9 methyltransferases in the absence of endogenous HP1γ, the cells were transiently transfected with a *Cre* recombinase expression plasmid. Western blot analysis showed that HP1γ mutants in which the chromo, chromo shadow, or hinge domain was deleted lost its ability to retain high levels of pluripotency markers and the proper expression of H3K9 methyltransferases (Figures 4B and 4F). Consistently, these mutants nearly abolished the ability to maintain global H3K9me2/3 levels. In contrast, the N- or C-terminus of HP1γ was dispensable for the expression of H3K9 methyltransferases and thus retained H3K9me2/3 levels as effectively as the wild-type. Notably, an HP1γ mutant harboring the V32M substitution in the chromo domain that abolishes the binding affinity to H3K9 methylation (Bannister et al., 2001; Yi et al., 2020) retained its ability to rescue the defects observed in $Hp1\alpha/\beta/\gamma^{\Delta/\Delta}$ cells, suggesting the presence of redundant or compensatory mechanisms. Overall, these results suggest that the known domains are essential for HP1γ-mediated expression of H3K9 methyltransferases and indicate that these regions confer upon HP1γ the ability to maintain the identity of ESCs.

Setdb1-deficient cells phenotypically resemble ESCs lacking the *Hp1* family

To systematically address potential functions of H3K9 methylation systems in ESCs, we generated a suite of single and multiple gene knockout mutants for the five members of the SET-domain H3K9 lysine methyltransferases using the CRISPR/Cas9 technology (Figures 5A and S3A–S3D). Whereas loss of *G9a* induced a reduced self-renewal and proliferation of ESCs, no obvious proliferation defects were observed when *Glp* was deleted. Although *G9a*- or *Glp*-deficient ESCs maintained an undifferentiated state as characterized by their colony morphology, strong staining for AP activity, and expression of the pluripotency markers (OCT4, SOX2, and NANOG) (Figures 5A–5C), teratomas derived from them featured a striking paucity of mature elements (Figure 5D), suggesting that $G9a^{\Delta/\Delta}$ or $Glp^{\Delta/\Delta}$ ESCs

Figure 4. The known domains of HP1 are essential for maintaining ESC pluripotency

(A) A schematic diagram showing the structures of wild-type and mutant HP1γ (left). The thin bent lines refer to deleted regions. A concise summary represents the ability of various mutants to rescue growth defect upon *Hp1* ablation (right).

(B) Western blot demonstrating the expression levels of different HP1γ mutants and pluripotency factors in cells of indicated genotypes. ACTIN was used as a loading control.

(C) Representative phase contrast images (top) and AP staining images (bottom) of ESC colonies of indicated genotypes on MEF feeders. Scale bar, 100 μm.

(D) Quantitation of colony types formed by cell lines shown in (C). AP-stained colonies were scored in three categories: undifferentiated, mixed (partially differentiated) and differentiated. Data are presented as the means ± SD of three independent experiments. $^{***}p < 0.001$ (Student t test) compared with the control.

(E) Growth curve of ESCs of indicated genotypes.

(F) Western blot demonstrating the changes in the levels of selected germ layer markers and histone modifications in ESCs of indicated genotypes. H3 and ACTIN were taken as loading controls.

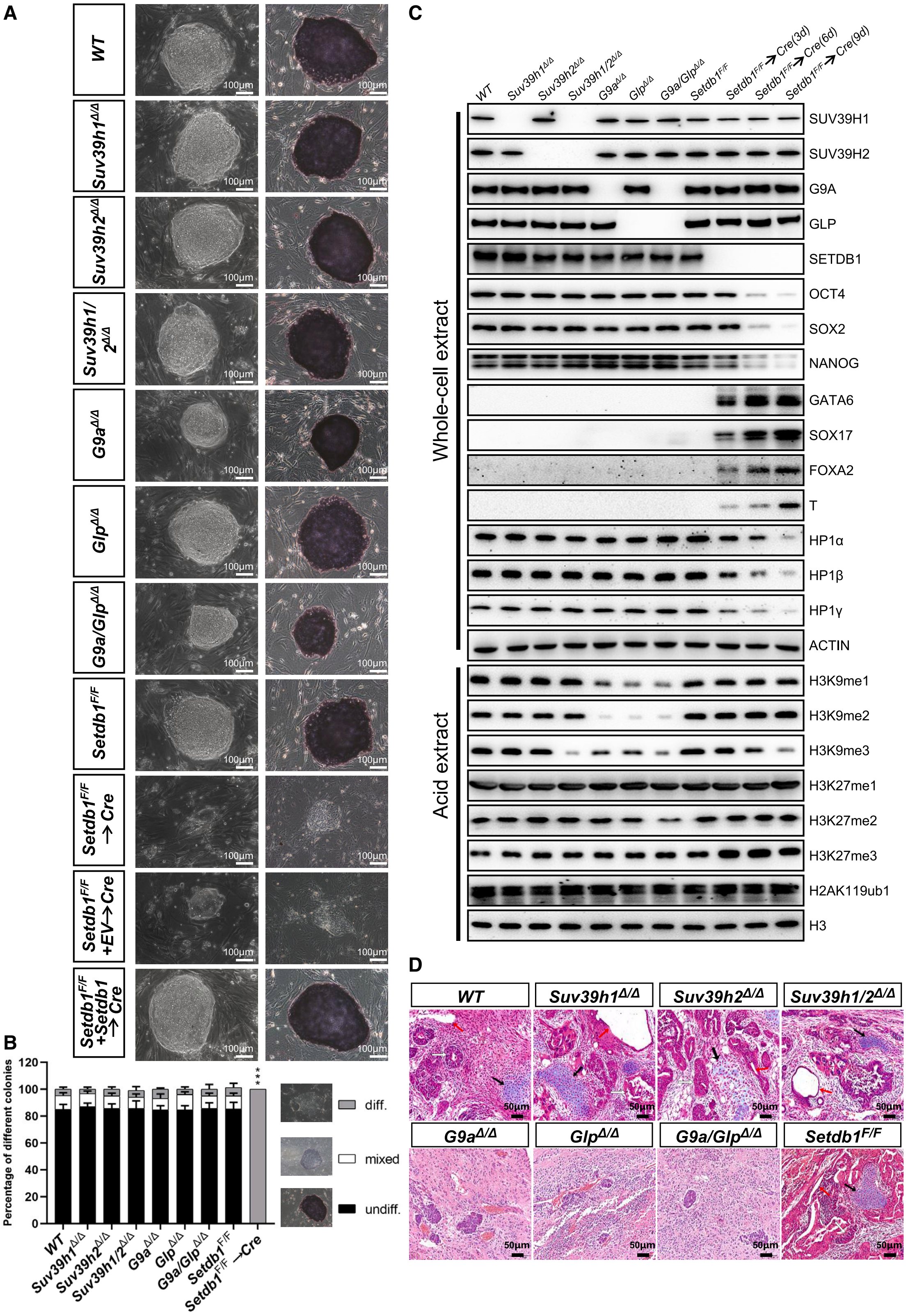

(legend on next page)

are significantly impaired in their ability to differentiate properly. In contrast, the *Suv39h1* and *Suv39h2* single or double mutant ESCs displayed no obvious phenotypic abnormalities as shown by colony morphology, pluripotent marker expression, and the formation of three germ layer teratomas *in vivo* (Figures 5A–5D).

To circumvent the lethality associated with complete loss of *Setdb1*, we engineered ESCs by inserting *loxP* sites in introns between exons 10 and 14 of the *Setdb1* gene, which was designed to remove exons 11–13 after Cre-mediated recombination to inactivate the gene. *Setdb1*$^{F/F}$ displayed normal SETDB1 protein levels and formed colonies and teratomas that were phenotypically identical to *wild-type* ESCs (Figures 5A–5D). However, as shown in Figure 5A, in contrast with the *Setdb1*$^{F/F}$, deletion of *Setdb1* resulted in flat and spreading colonies with irregular edges. Consistently, *Setdb1*$^{\Delta/\Delta}$ cells were AP negative and exhibited decreased expression levels of OCT4, SOX2, and NANOG, comparable with control cells, as well as strong expression of three germ layer markers, indicating that *Setdb1* loss severely impairs the self-renewal and pluripotency of ESCs (Figures 5A–5D). Consistent with the similar phenotypes of *Hp1*$\alpha/\beta/\gamma^{\Delta/\Delta}$ and *Setdb1*$^{\Delta/\Delta}$ cells, transcriptional analysis indicate that a large number of the genes deregulated in *Hp1*$\alpha/\beta/\gamma^{\Delta/\Delta}$ cells were also observed in ESCs deficient for *Setdb1* (Table S4), but not for *Suv39h1*, *Suv39h2*, *G9a*, or *Glp* (Mayer et al., 2020; Tatsumi et al., 2018) (Figure 6A). By analyzing publicly available ChIP-seq data for HP1 and SETDB1, we found that significant enrichment of HP1γ, but not HP1α and HP1β, were observed at SETDB1-bound genes (Figure S4A). A GO analysis revealed that HP1γ-SETDB1 co-bound genes were strongly associated with germ-line development (meiosis and spermatogenesis). The genomic snapshots presented in Figure S4B highlight the direct association of HP1γ and SETDB1 to selected germ cell-specific genes and also show co-enrichment with H3K9me3, but not H3K9me1, at these genes. Thus, the function of HP1 in maintaining ESC identity seems to be mediated by keeping the requisite levels of SETDB1. Interestingly, a significant and marked decrease in the expression levels of *Hp1* was observed in *Setdb1*$^{\Delta/\Delta}$ cells, suggesting a cross-positive feedback loop between the expression of *Hp1* and *Setdb1* (Figures 5C, S5A and S5B). Of note, the phenotypes observed in *Setdb1*$^{\Delta/\Delta}$ cells were specific to *Setdb1* deficiency, since they were fully restored by the introduction of a transgenic copy of wild-type *Setdb1* (Figure 5A).

We then examined the contribution of these H3K9 methyltransferases to the total levels of H3K9 and H3K27 methylation (Figure 5C). Western blot showed that *Suv39h1/2* single mutant ESCs did not exhibit a significant reduction in overall H3K9 and H3K27 methylation levels. By contrast, *Suv39h1/2* double deficient cells had greatly lowered H3K9me3. A substantial reduction of H3K9me2 and a partial reduction of H3K9me1, but only a modest reduction in H3K9me3, were observed in ESCs null for either *G9a* or *Glp*. Strikingly, double knockout of *G9a/Glp* further reduced the levels of H3K9me3, but not H3K9me1 or H3K9me2. Additionally, ablation of *Setdb1* gene in ESCs resulted in a partial reduction in total H3K9me3 and a substantial increase in total H3K27me3 (Figure 5C). Thus, the different H3K9 methyltransferases have distinct functions in establishing H3K9 and H3K27 methylation states, in agreement with previous studies (Padeken et al., 2022; Rea et al., 2000; Tachibana et al., 2008).

To determine to what extent the expression levels of H3K9 methyltransferases are needed to produce the observed defects in *Hp1*$\alpha/\beta/\gamma^{\Delta/\Delta}$ cells, we rescued *Hp1*$\alpha/\beta/\gamma^{\Delta/\Delta}$ ESCs by introducing the transgenes of H3K9 methyltransferases (Figure 6B). Introducing *G9a*, *Glp*, *Suv39h1*, and *Suv39h2* did not rescue the self-renewal defect observed in *Hp1*$\alpha/\beta/\gamma^{\Delta/\Delta}$ ESCs. In contrast, the introduced *Setdb1* cDNA could rescue the defects caused by *Hp1* deficiency in ESCs (Figures 6B–6D). The SETDB1-expressing *Hp1*-null ESCs were viable and retain their undifferentiated state as characterized by tightly packed morphology; AP staining; high levels of the core pluripotency factors OCT4, SOX2, and NANOG; and no precocious expression of germ layer-specific genes. Surprisingly, ectopic expression of *Setdb1* did not rescue the H3K9me2/3 level of *Hp1*$\alpha/\beta/\gamma^{\Delta/\Delta}$ cells (Figures 6D and S5C), indicating that HP1 is required for H3K9 methyltransferase-mediated establishment of H3K9me2/3. Furthermore, these rescued cells formed teratomas after being injected into nude mice. In histologic analyses, the most abundant components of SET DB1-expressing *Hp1*-null teratomas were undifferentiated areas and featured a striking paucity of mature elements derived from three germ layers (Figure 6E). Interestingly, the

Figure 5. *Setdb1*-deficient cells phenotypically resemble ESCs lacking the *Hp1* family

(A) Representative phase contrast images (left) and AP staining images (right) of ESC colonies of indicated genotypes on MEF feeders. Scale bar, 100 μm.

(B) Quantitation of colony types formed by cell lines shown in (A). AP-stained colonies were scored in three categories: undifferentiated, mixed (partially differentiated) and differentiated. Data are presented as the means ± SD of three independent experiments. ***$p < 0.001$ (Student t test) compared with the control.

(C) Western blot analysis of indicated cells using antibodies indicated on the right.

(D) Histological sections of hematoxylin and eosin-stained teratomas of indicated genotypes performed. Neural rosette (ectoderm; white arrow), cartilage (mesoderm; black arrow), and gut epithelium (endoderm; red arrow) are shown. Scale bar, 50 μm. See also Figure S3.

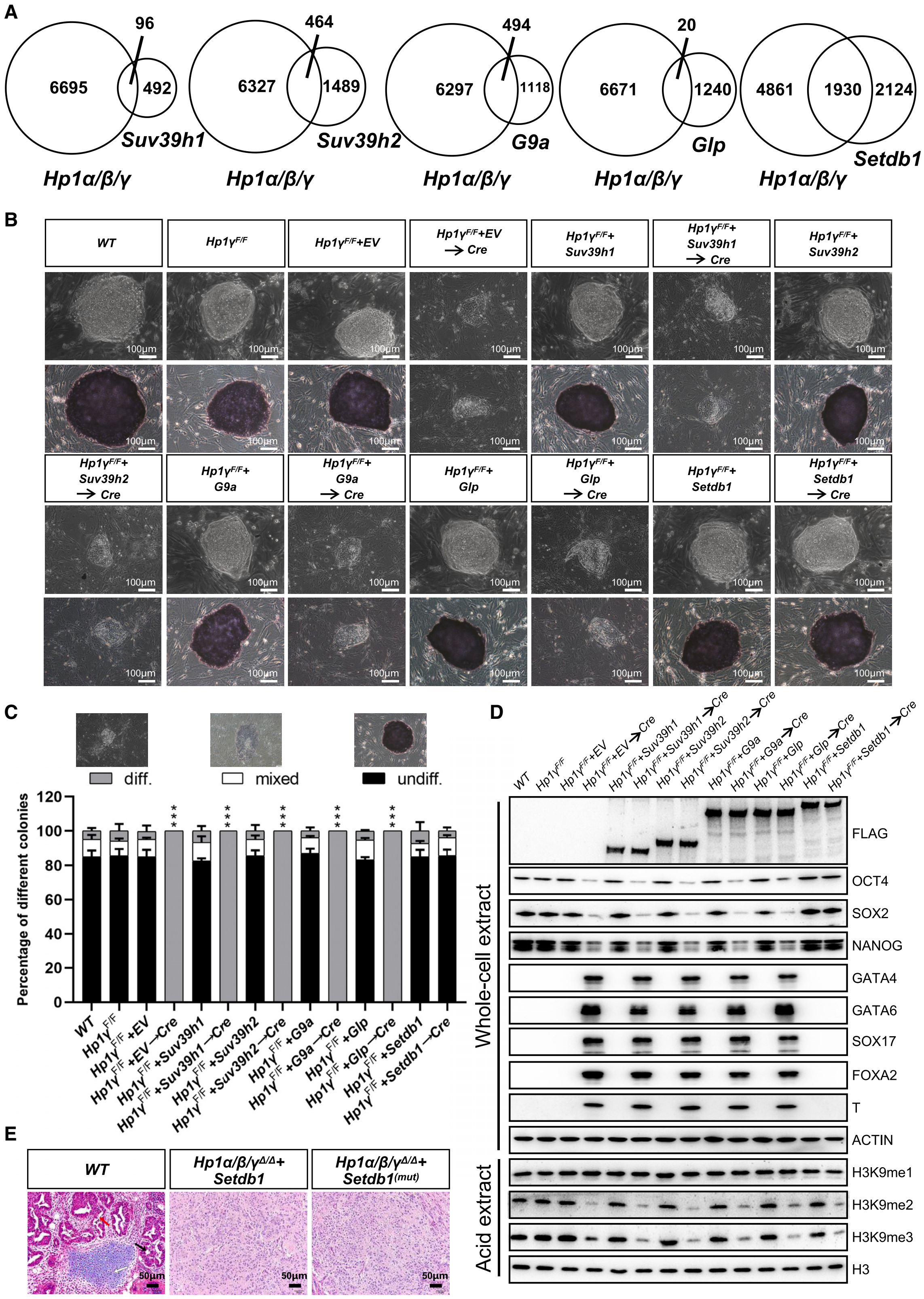

(legend on next page)

detrimental phenotype of *Hp1*-null ESCs could also be rescued by lysine methyltransferase-defective (C1243A) *Setdb1* (Matsui et al., 2010), suggesting that the catalytic activity of SETDB1 is not required for its ability to rescue defects associated with *Hp1* deficiency in ESCs (Figures S5D–S5F). Together, these findings reveal that HP1 sustains high expression levels of H3K9 methyltransferases, particularly SETDB1, that in turn directly preserves ESC identity.

Forced *Oct4* or *Nanog* expression renders ESC self-renewal independent of the HP1 family

Previous studies have demonstrated that SETDB1 is interactive with the major pluripotent system containing OCT4 and NANOG to guard ESC identity (Loh et al., 2006; Yuan et al., 2009). Additionally, ChIP-seq analysis identified *Nanog* as a direct target of SETDB1 and OCT4, and revealed significant binding of SETDB1, OCT4, and NANOG to the promoter regions of *Hp1β* and *Hp1γ* (Figure 7A). We, therefore, hypothesized that the feedback regulatory loop comprising HP1 and SETDB1 may interplay with OCT4 and NANOG to control ESC pluripotency. The above-mentioned $Hp1\alpha/\beta^{\Delta/\Delta};Hp1\gamma^{F/F}$ ESCs were used to address this hypothesis. Although introducing *Sox2* did not affect *Hp1*-null ESCs, forced *Nanog* or *Oct4* expression prevented the decline in pluripotency protein levels observed in $Hp1\alpha/\beta/\gamma^{\Delta/\Delta}$ and produced viable colonies (Figures 7B–7D). The *Nanog*- or *Oct4*-expressing *Hp1*-null ESCs were able to expand continuously for at least 30 passages without any change in colony morphology. These rescued cells were positive for AP, OCT4, NANOG, and SOX2. Intriguingly, expression levels of H3K9 methyltransferases SUV39H1/2, G9A, GLP, and SET DB1 in *Nanog*- or *Oct4*-rescued cells were comparable with those in $Hp1\alpha/\beta^{\Delta/\Delta};Hp1\gamma^{F/F}$ cells, suggesting that *Nanog* and *Oct4* are able to rescue the growth defect in $Hp1\alpha/\beta/\gamma^{\Delta/\Delta}$ cells by regulating the expression levels of H3K9 methyltransferases (Figure 7D). Like *Setdb1*, forced expression of *Nanog* or *Oct4* did not restore the H3K9me2/3 levels in $Hp1\alpha/\beta/\gamma^{\Delta/\Delta}$ cells, further indicating that *Hp1* is essential for H3K9 methyltransferase-mediated establishment and maintenance of H3K9me2/3. Similar to teratomas from *Setdb1*-expressing *Hp1*-null ESCs, the teratomas derived from *Nanog*- or *Oct4*-rescued *Hp1*-null ESCs were characterized by an almost complete absence of differentiated tissues derived from the three germ layers (Figure 7E). The loss of differentiation capacity caused by overexpression of *Oct4* and *Nanog* may account for the paucity of mature elements in the *Nanog*- or *Oct4*-rescued *Hp1*-null teratomas. These results might indicate that OCT4 and NANOG are critical downstream effectors of the maintenance of ESC identity orchestrated by HP1 family (Figure 7F).

DISCUSSION

HP1 is an established reader protein of H3K9me2/3 (James and Elgin, 1986; Padeken et al., 2022). Despite our comprehensive knowledge of the HP1 family in heterochromatin formation and epigenetic gene silencing, its role in maintaining the pluripotent state of ESCs remains largely uncharacterized. The mild phenotypes of mice lacking individual of *Hp1* family members suggest redundancy among members of the family (Abe et al., 2011; Aucott et al., 2008). Interestingly, recent reports have demonstrated that HP1 paralogs act redundantly to ensure ESC viability by maintaining protein stability of H3K9 methyltransferases (Maeda and Tachibana, 2022). We established and examined the phenotypes of ESCs lacking one or two *Hp1* paralogs in any combinations of *Hp1* family members to assess possible functional redundancies within the gene family. Whereas the single mutant ESCs developed no obvious phenotype, the double mutants exhibited impaired self-renewal and altered differentiation toward endodermal lineage. Importantly, complete loss of all three *Hp1* paralogs resulted in the loss of self-renewal and pluripotency maintenance ability in ESCs, which was accompanied by decreased levels of H3K9 methyltransferases with subsequent down-regulation of global H3K9me2/3 (Figure 1). In addition, ablation of *Setdb1* phenocopied the loss of *Hp1* family in ESCs and caused a dramatic decrease in *Hp1* expression (Figure 5). Together, our data

Figure 6. Introduction of *Setdb1* largely rescues the defects caused by *Hp1* deficiency in ESCs

(A) Venn diagrams showing the significant overlapping of differentially expressed genes in ESCs of indicated genotypes. Published RNA-seq data were acquired from the NCBI (GEO: GSE131015, GSE102423, and GSE99155).

(B) Representative phase contrast images (top) and AP staining images (bottom) of ESC colonies of indicated genotypes on MEF feeders. Scale bar, 100 μm.

(C) Quantitation of colony types formed by cell lines shown in (A). AP-stained colonies were scored in three categories: undifferentiated, mixed (partially differentiated) and differentiated. Data are presented as the means ± SD of three independent experiments. ***$p < 0.001$ (Student t test) compared with the control.

(D) Western blot demonstrating the expression levels of different H3K9 methyltransferases, pluripotency factors, germ layer markers, and histone modifications in cells of indicated genotypes. ACTIN and H3 were taken as the loading controls.

(E) Histological sections of hematoxylin and eosin-stained teratomas of indicated genotypes performed. Neural rosette (ectoderm; white arrow), cartilage (mesoderm; black arrow), and gut epithelium (endoderm; red arrow) are shown. Scale bar, 50 μm. See also Figures S4 and S5.

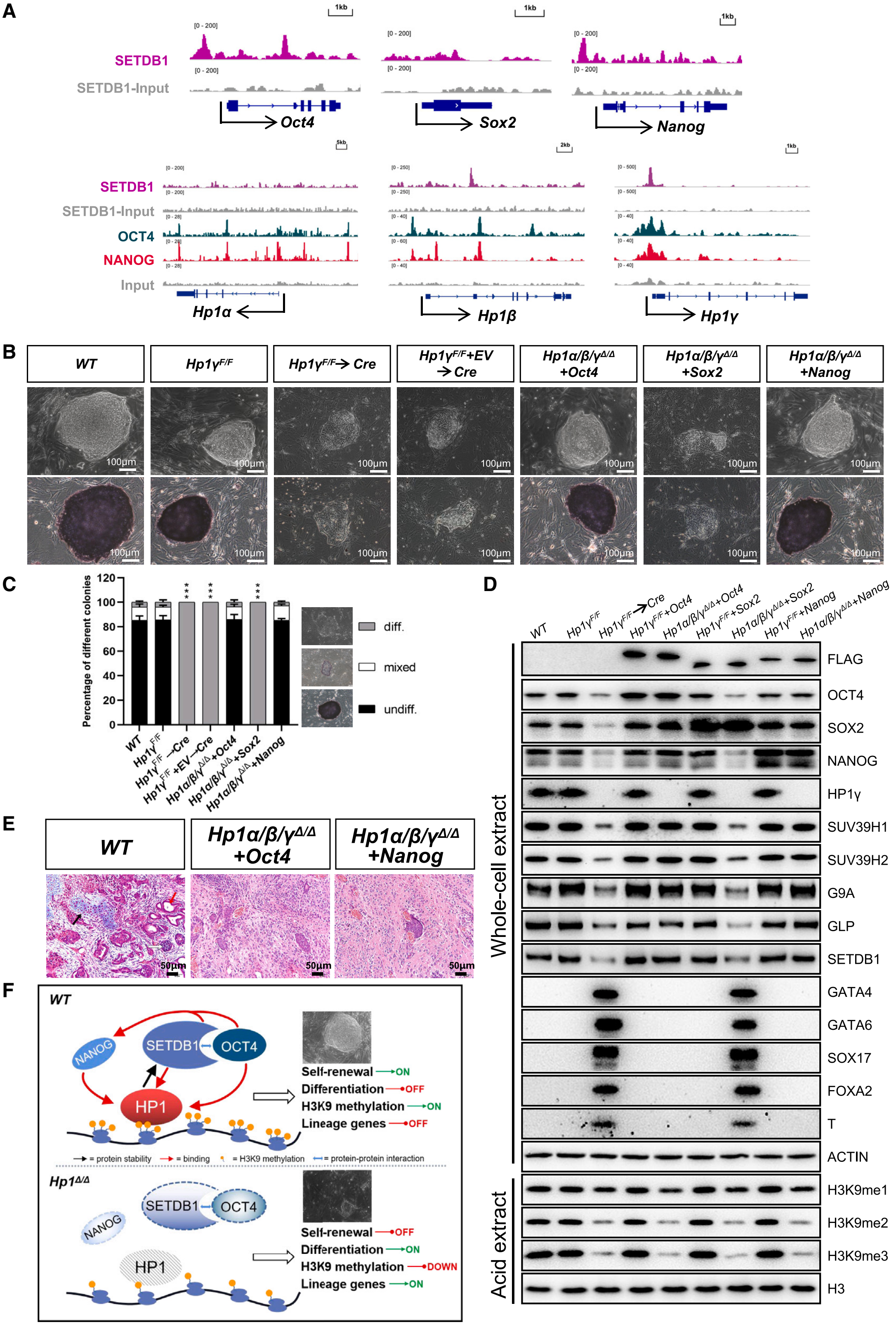

(legend on next page)

support that crosstalks exist between the H3K9 methylation writers and their reader HP1 that contribute to both the maintenance of pluripotent state and the transcriptional repression of developmentally regulated genes in ESCs (Figure 7F).

Despite their redundancy, genetic disruption of an individual H3K9 methyltransferase results in the leakage expression of lineage-inappropriate genes, impaired cell differentiation and loss of tissue identity (Dodge et al., 2004; Peters et al., 2001; Tachibana et al., 2002). RT-qPCR demonstrated that *Hp1* ablation elicits delayed decreases in mRNA steady-state levels of these methyltransferases in ESCs (Figure S1E). In contrast, the loss of HP1 activity in ESCs led to an almost immediate down-regulation of protein levels in H3K9 methyltransferases, particularly SETDB1 (Figure 1K), suggesting a contribution of post-transcriptional events. Additionally, ectopic expression of *Setdb1* can rescue the proliferative defects in *Hp1*-null ESCs (Figure 6). Similarly, HP1 was previously reported to specifically maintain protein stability of H3K9 methyltransferases by tethering these enzymes to chromatin (Maeda and Tachibana, 2022). Therefore, the ability of HP1 to maintain the gene expression of H3K9 methyltransferases in ESCs is mainly due to a post-transcriptional effect.

OCT4, SOX2, and NANOG have been established as the core pluripotency factors that form a transcriptional circuitry to induce the expression of pluripotency related genes and to repress developmental genes (Boyer et al., 2005). Previous study has revealed that SETDB1 interacts with OCT4, which in turn recruits SETDB1 to silence lineage-specific genes (Yuan et al., 2009). In addition, *Setdb1* is also a target of NANOG in ESCs (Loh et al., 2006), further implicating its functional importance in ESC biology. Importantly, deletion of *Setdb1* resulted in a dramatic decrease in expression of *Oct4*, *Sox2*, and *Nanog* and spontaneous differentiation of ESCs, a phenotype that is similar to deletion of all three *Hp1* isoforms. Moreover, ChIP-seq analysis revealed the presence of SETDB1 and OCT4 at the promoter regions of *Nanog* and the direct association of SETDB1, OCT4, and NANOG to *Hp1* (Figure 7A). These results allow us to envision a model in which the regulatory loop comprising HP1 and SETDB1 is integrated with the major pluripotent transcription factors OCT4 and NANOG to safeguard ESC identity (Figure 7F). This is consistent with our findings as we demonstrated that *Setdb1*, *Oct4*, and *Nanog* were largely able to rescue the *Hp1* triple mutant defects (Figures 6 and 7). The teratomas derived from the rescued cells, however, were primitive with poorly differentiated elements, indicating their impaired pluripotency. This might be caused by the low levels of H3K9me2/3 in the rescued cells. The impact of H3K9me2/3 on the essential cellular functions such as pluripotency and lineage-specific differentiation in ESCs, as well as the underlying molecular mechanism, is under investigation.

The identification of HP1s and H3K9 methylation systems as being key regulators in the molecular circuitry of pluripotency will allow the pursuit of a more comprehensive approach to better understanding the biology of ESCs. Despite the evidences found in our study in favor of an unanticipated crosstalk between H3K9 methyltransferases and their reader HP1 in safeguarding ESC identity, direct proof of a functional interplay between them merits further investigation. In summary, our findings provide strong evidence that the HP1 family redundantly maintains the self-renewal and pluripotency of ESCs by regulating protein stability of H3K9 methyltransferases, particularly SETDB1, which in turn sustain the expression of *Oct4* and *Nanog*. Since H3K9 methylation is essential for normal embryonic development and tissue homeostasis, understanding to what extent the HP1-mediated control of H3K9 methyltransferase activity is relevant in other stem cell types remains to be determined in future investigations (Nicetto and Zaret, 2019; Padeken et al., 2022). In addition, the ability of H3K9 methylation to influence cell identity challenges the established concept of H3K9me3-decorated heterochromatin as compacted, transcriptionally repressed chromatin and affords a further level of understanding of the coordination of H3K9 methylation with cell fate determination.

Figure 7. Forced *Nanog* or *Oct4* expression renders ESC self-renewal independent of *Hp1* family

(A) Representative ChIP-seq tracks for SETDB1, OCT4, and NANOG at selected target gene loci in *wild-type* ESCs. Published ChIP-seq data for SETDB1, OCT4, and NANOG were obtained from the NCBI GEO database (GEO: GSE126243 and GSE129721).

(B) Representative phase contrast images (top) and AP staining images (bottom) of ESC colonies of indicated genotypes on MEF feeders. Scale bar, 100 μm.

(C) Quantitation of colony types formed by cell lines shown in (B). AP-stained colonies were scored in three categories: undifferentiated, mixed (partially differentiated) and differentiated. Data are presented as the means ± SD of three independent experiments. ***$p < 0.001$ (Student t test) compared with the controls.

(D) Western blot demonstrating the expression levels of ectopic and endogenous pluripotency factors, H3K9 methyltransferases, germ layer markers and histone modifications in cells of indicated genotypes. ACTIN and H3 were taken as the loading controls.

(E) Histological sections of hematoxylin and eosin-stained teratomas of indicated genotypes performed. Neural rosette (ectoderm; white arrow), cartilage (mesoderm; black arrow), and gut epithelium (endoderm; red arrow) are shown. Scale bar, 50 μm. Results shown are representative of five injected mice.

(F) Schematic representing of the role of HP1 family in the maintenance of ESC pluripotency.

EXPERIMENTAL PROCEDURES

Resource availability

Corresponding author

Further information and requests for resources and reagents should be directed to and will be fulfilled by the lead contact, Jinzhong Qin (qinjz@nju.edu.cn).

Materials availability

Cell lines generated in this study are available from the lead contact upon request.

Data and code availability

The RNA-seq data have been deposited at GEO: GSE210606 and are publicly available as of the date of publication. This paper does not report original code.

Mice

The experimental animal facility has been accredited by the Association for Assessment and Accreditation of Laboratory Animal Care International, and all the experimental procedures involving animals were performed with the approval of the Institutional Animal Care and Use Committee of the Model Animal Research Center of Nanjing University. SCID mice were housed in autoclaved microisolator cages under specific pathogen-free conditions, and all food, water, and bedding were autoclaved before use.

Cell lines

ESCs were routinely cultured on mitotically inactivated MEF feeder layers in gelatin-coated dishes with DMEM (15% fetal bovine serum [Gibco], 100 U/mL penicillin-streptomycin [Thermo Fisher], 1,000 U/mL LIF, 2 mM L-glutamine [Invitrogen], 0.5 mM β-mercaptoethanol [Gibico], and 1:100 non-essential amino acid [Gibico]). ESCs were separated from MEF feeder cells by trypsinization, followed by incubation on gelatin-coated dishes for 30 min. Non-adherent cells consisting mainly of ESCs were used for further experiments. The culture medium of HEK293FT and MEFs was DMEM added with 10% fetal bovine serum and 100 U/mL penicillin-streptomycin. MEFs were derived from 13.5 dpc embryos using standard procedures (Qin et al., 2012). All cell lines were cultured at 37°C and 5% CO_2.

Quantification and statistical analysis

Unless stated otherwise, all data were presented as mean values ± SD or mean values ± SEM for experiments conducted with at least three independent experiments. GraphPad Prism 5 was used to analyze data and perform statistical tests and generate p values using two-tailed the Student t test for comparisons between two datasets, and p values of less than 0.05 were considered to be statistically significant. Statistical significance was presented in figures in the following manner: $*p < 0.05$, $**p < 0.01$, $***p < 0.001$.

SUPPLEMENTAL INFORMATION

Supplemental information can be found online at https://doi.org/10.1016/j.stemcr.2023.08.004.

AUTHOR CONTRIBUTIONS

Conceptualization, J.Q.; methodology, L.D., H.L., L.Z., J.W., C.W., and S.L.; formal analysis, L.D., J.W.,C.W., L.X., and J.Q.; mechanistic investigations, L.D., H.L., B.W., Y.S., C.W., Y.X., S.L., X.L., and J.Q.; writing – original draft, L.D. and J.Q.; and writing – review and editing, L.D. and J.Q.; funding aAcquisition, J.Q.; supervision, J.Q.

ACKNOWLEDGMENTS

We are indebted to all members of the Qin laboratory and to Yikai Huang and Ting Su for experimental advice and helpful discussions. This work was supported by grants from the National Natural Science Foundation of China (31970810) to J.Q. and by the Fundamental Research Funds for the Central Universities (021414380533).

CONFLICT OF INTERESTS

The authors declare no competing interests.

Received: February 15, 2023
Revised: August 7, 2023
Accepted: August 7, 2023
Published: August 31, 2023

REFERENCES

Abe, K., Naruse, C., Kato, T., Nishiuchi, T., Saitou, M., and Asano, M. (2011). Loss of heterochromatin protein 1 gamma reduces the number of primordial germ cells via impaired cell cycle progression in mice. Biol. Reprod. *85*, 1013–1024. https://doi.org/10.1095/biolreprod.111.091512.

Aucott, R., Bullwinkel, J., Yu, Y., Shi, W., Billur, M., Brown, J.P., Menzel, U., Kioussis, D., Wang, G., Reisert, I., et al. (2008). HP1-beta is required for development of the cerebral neocortex and neuromuscular junctions. J. Cell Biol. *183*, 597–606. https://doi.org/10.1083/jcb.200804041.

Bannister, A.J., Zegerman, P., Partridge, J.F., Miska, E.A., Thomas, J.O., Allshire, R.C., and Kouzarides, T. (2001). Selective recognition of methylated lysine 9 on histone H3 by the HP1 chromo domain. Nature *410*, 120–124. https://doi.org/10.1038/35065138.

Bilodeau, S., Kagey, M.H., Frampton, G.M., Rahl, P.B., and Young, R.A. (2009). SetDB1 contributes to repression of genes encoding developmental regulators and maintenance of ES cell state. Genes Dev. *23*, 2484–2489. https://doi.org/10.1101/gad.1837309.

Boyer, L.A., Lee, T.I., Cole, M.F., Johnstone, S.E., Levine, S.S., Zucker, J.P., Guenther, M.G., Kumar, R.M., Murray, H.L., Jenner, R.G., et al. (2005). Core transcriptional regulatory circuitry in human embryonic stem cells. Cell *122*, 947–956. https://doi.org/10.1016/j.cell.2005.08.020.

Canzio, D., Liao, M., Naber, N., Pate, E., Larson, A., Wu, S., Marina, D.B., Garcia, J.F., Madhani, H.D., Cooke, R., et al. (2013). A conformational switch in HP1 releases auto-inhibition to drive heterochromatin assembly. Nature *496*, 377–381. https://doi.org/10.1038/nature12032.

Chen, J., Liu, H., Liu, J., Qi, J., Wei, B., Yang, J., Liang, H., Chen, Y., Chen, J., Wu, Y., et al. (2013). H3K9 methylation is a barrier during somatic cell reprogramming into iPSCs. Nat. Genet. *45*, 34–42. https://doi.org/10.1038/ng.2491.

Cowieson, N.P., Partridge, J.F., Allshire, R.C., and McLaughlin, P.J. (2000). Dimerisation of a chromo shadow domain and distinctions from the chromodomain as revealed by structural analysis. Curr. Biol. *10*, 517–525. https://doi.org/10.1016/s0960-9822(00)00467-x.

Dodge, J.E., Kang, Y.K., Beppu, H., Lei, H., and Li, E. (2004). Histone H3-K9 methyltransferase ESET is essential for early development. Mol. Cell Biol. *24*, 2478–2486. https://doi.org/10.1128/mcb.24.6.2478-2486.2004.

Eissenberg, J.C., James, T.C., Foster-Hartnett, D.M., Hartnett, T., Ngan, V., and Elgin, S.C. (1990). Mutation in a heterochromatin-specific chromosomal protein is associated with suppression of position-effect variegation in Drosophila melanogaster. Proc. Natl. Acad. Sci. USA *87*, 9923–9927. https://doi.org/10.1073/pnas.87.24.9923.

Endoh, M., Endo, T.A., Shinga, J., Hayashi, K., Farcas, A., Ma, K.W., Ito, S., Sharif, J., Endoh, T., Onaga, N., et al. (2017). PCGF6-PRC1 suppresses premature differentiation of mouse embryonic stem cells by regulating germ cell-related genes. Elife *6*, e21064. https://doi.org/10.7554/eLife.21064.

Gao, Z., Zhang, J., Bonasio, R., Strino, F., Sawai, A., Parisi, F., Kluger, Y., and Reinberg, D. (2012). PCGF homologs, CBX proteins, and RYBP define functionally distinct PRC1 family complexes. Mol. Cell *45*, 344–356. https://doi.org/10.1016/j.molcel.2012.01.002.

Hisada, K., Sánchez, C., Endo, T.A., Endoh, M., Román-Trufero, M., Sharif, J., Koseki, H., and Vidal, M. (2012). RYBP represses endogenous retroviruses and preimplantation- and germ line-specific genes in mouse embryonic stem cells. Mol. Cell Biol. *32*, 1139–1149. https://doi.org/10.1128/mcb.06441-11.

Jacobs, S.A., and Khorasanizadeh, S. (2002). Structure of HP1 chromodomain bound to a lysine 9-methylated histone H3 tail. Science *295*, 2080–2083. https://doi.org/10.1126/science.1069473.

James, T.C., and Elgin, S.C. (1986). Identification of a nonhistone chromosomal protein associated with heterochromatin in Drosophila melanogaster and its gene. Mol. Cell Biol. *6*, 3862–3872. https://doi.org/10.1128/mcb.6.11.3862-3872.1986.

Loh, Y.H., Wu, Q., Chew, J.L., Vega, V.B., Zhang, W., Chen, X., Bourque, G., George, J., Leong, B., Liu, J., et al. (2006). The Oct4 and Nanog transcription network regulates pluripotency in mouse embryonic stem cells. Nat. Genet. *38*, 431–440. https://doi.org/10.1038/ng1760.

Maeda, I., Okamura, D., Tokitake, Y., Ikeda, M., Kawaguchi, H., Mise, N., Abe, K., Noce, T., Okuda, A., and Matsui, Y. (2013). Max is a repressor of germ cell-related gene expression in mouse embryonic stem cells. Nat. Commun. *4*, 1754. https://doi.org/10.1038/ncomms2780.

Maeda, R., and Tachibana, M. (2022). HP1 maintains protein stability of H3K9 methyltransferases and demethylases. EMBO Rep. *23*, e53581. https://doi.org/10.15252/embr.202153581.

Matsui, T., Leung, D., Miyashita, H., Maksakova, I.A., Miyachi, H., Kimura, H., Tachibana, M., Lorincz, M.C., and Shinkai, Y. (2010). Proviral silencing in embryonic stem cells requires the histone methyltransferase ESET. Nature *464*, 927–931. https://doi.org/10.1038/nature08858.

Mayer, D., Stadler, M.B., Rittirsch, M., Hess, D., Lukonin, I., Winzi, M., Smith, A., Buchholz, F., and Betschinger, J. (2020). Zfp281 orchestrates interconversion of pluripotent states by engaging Ehmt1 and Zic2. EMBO J. *39*, e102591. https://doi.org/10.15252/embj.2019102591.

Nicetto, D., Donahue, G., Jain, T., Peng, T., Sidoli, S., Sheng, L., Montavon, T., Becker, J.S., Grindheim, J.M., Blahnik, K., et al. (2019). H3K9me3-heterochromatin loss at protein-coding genes enables developmental lineage specification. Science *363*, 294–297. https://doi.org/10.1126/science.aau0583.

Nicetto, D., and Zaret, K.S. (2019). Role of H3K9me3 heterochromatin in cell identity establishment and maintenance. Curr. Opin. Genet. Dev. *55*, 1–10. https://doi.org/10.1016/j.gde.2019.04.013.

Nielsen, P.R., Nietlispach, D., Mott, H.R., Callaghan, J., Bannister, A., Kouzarides, T., Murzin, A.G., Murzina, N.V., and Laue, E.D. (2002). Structure of the HP1 chromodomain bound to histone H3 methylated at lysine 9. Nature *416*, 103–107. https://doi.org/10.1038/nature722.

Nozawa, R.S., Nagao, K., Masuda, H.T., Iwasaki, O., Hirota, T., Nozaki, N., Kimura, H., and Obuse, C. (2010). Human POGZ modulates dissociation of HP1alpha from mitotic chromosome arms through Aurora B activation. Nat. Cell Biol. *12*, 719–727. https://doi.org/10.1038/ncb2075.

Ogawa, H., Ishiguro, K.I., Gaubatz, S., Livingston, D.M., and Nakatani, Y. (2002). A complex with chromatin modifiers that occupies E2F- and Myc-responsive genes in G0 cells. Science *296*, 1132–1136. https://doi.org/10.1126/science.1069861.

Padeken, J., Methot, S.P., and Gasser, S.M. (2022). Establishment of H3K9-methylated heterochromatin and its functions in tissue differentiation and maintenance. Nat. Rev. Mol. Cell Biol. *23*, 623–640. https://doi.org/10.1038/s41580-022-00483-w.

Peters, A.H., O'Carroll, D., Scherthan, H., Mechtler, K., Sauer, S., Schöfer, C., Weipoltshammer, K., Pagani, M., Lachner, M., Kohlmaier, A., et al. (2001). Loss of the Suv39h histone methyltransferases impairs mammalian heterochromatin and genome stability. Cell *107*, 323–337. https://doi.org/10.1016/s0092-8674(01)00542-6.

Qin, J., Wang, C., Zhu, Y., Su, T., Dong, L., Huang, Y., and Hao, K. (2021). Mga safeguards embryonic stem cells from acquiring extraembryonic endoderm fates. Sci. Adv. *7*, eabe5689. https://doi.org/10.1126/sciadv.abe5689.

Qin, J., Whyte, W.A., Anderssen, E., Apostolou, E., Chen, H.H., Akbarian, S., Bronson, R.T., Hochedlinger, K., Ramaswamy, S., Young, R.A., and Hock, H. (2012). The polycomb group protein L3mbtl2 assembles an atypical PRC1-family complex that is essential in pluripotent stem cells and early development. Cell Stem Cell *11*, 319–332. https://doi.org/10.1016/j.stem.2012.06.002.

Rea, S., Eisenhaber, F., O'Carroll, D., Strahl, B.D., Sun, Z.W., Schmid, M., Opravil, S., Mechtler, K., Ponting, C.P., Allis, C.D., and Jenuwein, T. (2000). Regulation of chromatin structure by site-specific histone H3 methyltransferases. Nature *406*, 593–599. https://doi.org/10.1038/35020506.

Richards, E.J., and Elgin, S.C.R. (2002). Epigenetic codes for heterochromatin formation and silencing: rounding up the usual suspects. Cell *108*, 489–500. https://doi.org/10.1016/s0092-8674(02)00644-x.

Soufi, A., Donahue, G., and Zaret, K.S. (2012). Facilitators and impediments of the pluripotency reprogramming factors' initial engagement with the genome. Cell *151*, 994–1004. https://doi.org/10.1016/j.cell.2012.09.045.

Strahl, B.D., and Allis, C.D. (2000). The language of covalent histone modifications. Nature *403*, 41–45. https://doi.org/10.1038/47412.

Tachibana, M., Matsumura, Y., Fukuda, M., Kimura, H., and Shinkai, Y. (2008). G9a/GLP complexes independently mediate H3K9 and DNA methylation to silence transcription. EMBO J. *27*, 2681–2690. https://doi.org/10.1038/emboj.2008.192.

Tachibana, M., Sugimoto, K., Nozaki, M., Ueda, J., Ohta, T., Ohki, M., Fukuda, M., Takeda, N., Niida, H., Kato, H., and Shinkai, Y. (2002). G9a histone methyltransferase plays a dominant role in euchromatic histone H3 lysine 9 methylation and is essential for early embryogenesis. Genes Dev. *16*, 1779–1791. https://doi.org/10.1101/gad.989402.

Tatsumi, D., Hayashi, Y., Endo, M., Kobayashi, H., Yoshioka, T., Kiso, K., Kanno, S., Nakai, Y., Maeda, I., Mochizuki, K., et al. (2018). DNMTs and SETDB1 function as co-repressors in MAX-mediated repression of germ cell-related genes in mouse embryonic stem cells. PLoS One *13*, e0205969. https://doi.org/10.1371/journal.pone.0205969.

Yeap, L.S., Hayashi, K., and Surani, M.A. (2009). ERG-associated protein with SET domain (ESET)-Oct4 interaction regulates pluripotency and represses the trophectoderm lineage. Epigenet. Chromatin *2*, 12. https://doi.org/10.1186/1756-8935-2-12.

Yi, S.A., Lee, D.H., Kim, G.W., Ryu, H.W., Park, J.W., Lee, J., Han, J., Park, J.H., Oh, H., Lee, J., et al. (2020). HPV-mediated nuclear export of HP1γ drives cervical tumorigenesis by downregulation of p53. Cell Death Differ. *27*, 2537–2551. https://doi.org/10.1038/s41418-020-0520-5.

Yuan, P., Han, J., Guo, G., Orlov, Y.L., Huss, M., Loh, Y.H., Yaw, L.P., Robson, P., Lim, B., and Ng, H.H. (2009). Eset partners with Oct4 to restrict extraembryonic trophoblast lineage potential in embryonic stem cells. Genes Dev. *23*, 2507–2520. https://doi.org/10.1101/gad.1831909.

Zhao, W., Tong, H., Huang, Y., Yan, Y., Teng, H., Xia, Y., Jiang, Q., and Qin, J. (2017). Essential Role for Polycomb Group Protein Pcgf6 in Embryonic Stem Cell Maintenance and a Noncanonical Polycomb Repressive Complex 1 (PRC1) Integrity. J. Biol. Chem. *292*, 2773–2784. https://doi.org/10.1074/jbc.M116.763961.

Stem Cell Reports, Volume *18*

Supplemental Information

A functional crosstalk between the H3K9 methylation writers and their reader HP1 in safeguarding embryonic stem cell identity

Lixia Dong, Huaqi Liao, Linchun Zhao, Jingnan Wang, Congcong Wang, Bowen Wang, Yanqi Sun, Lijun Xu, Yin Xia, Shizhang Ling, Xin Lou, and Jinzhong Qin

Supplementary Materials for

A functional crosstalk between the H3K9 methylation writers and their reader HP1 in safeguarding embryonic stem cell identity

Lixia Dong,[1,6] Huaqi Liao,[1,6] Linchun Zhao,[1,6] Jingnan Wang,[1,6] Congcong Wang,[1] Bowen Wang,[1] Yanqi Sun,[1] Lijun Xu,[1] Yin Xia,[2] Shizhang Ling,[3,*] Xin Lou,[4,*] and Jinzhong Qin[1,5,7,*]

*Correspondence author. Jinzhong Qin, Email: qinjz@nju.edu.cn (J.Q.); Xin Lou, Email: xin.lou@zhejianglab.edu.cn(X.L.); Shizhang Ling, Email:lingsz@hotmail.com (S.L.)

The PDF file includes:

Figures. S1 to S5

Other Supplementary Material for this manuscript includes the following:
Tables S1 to S5

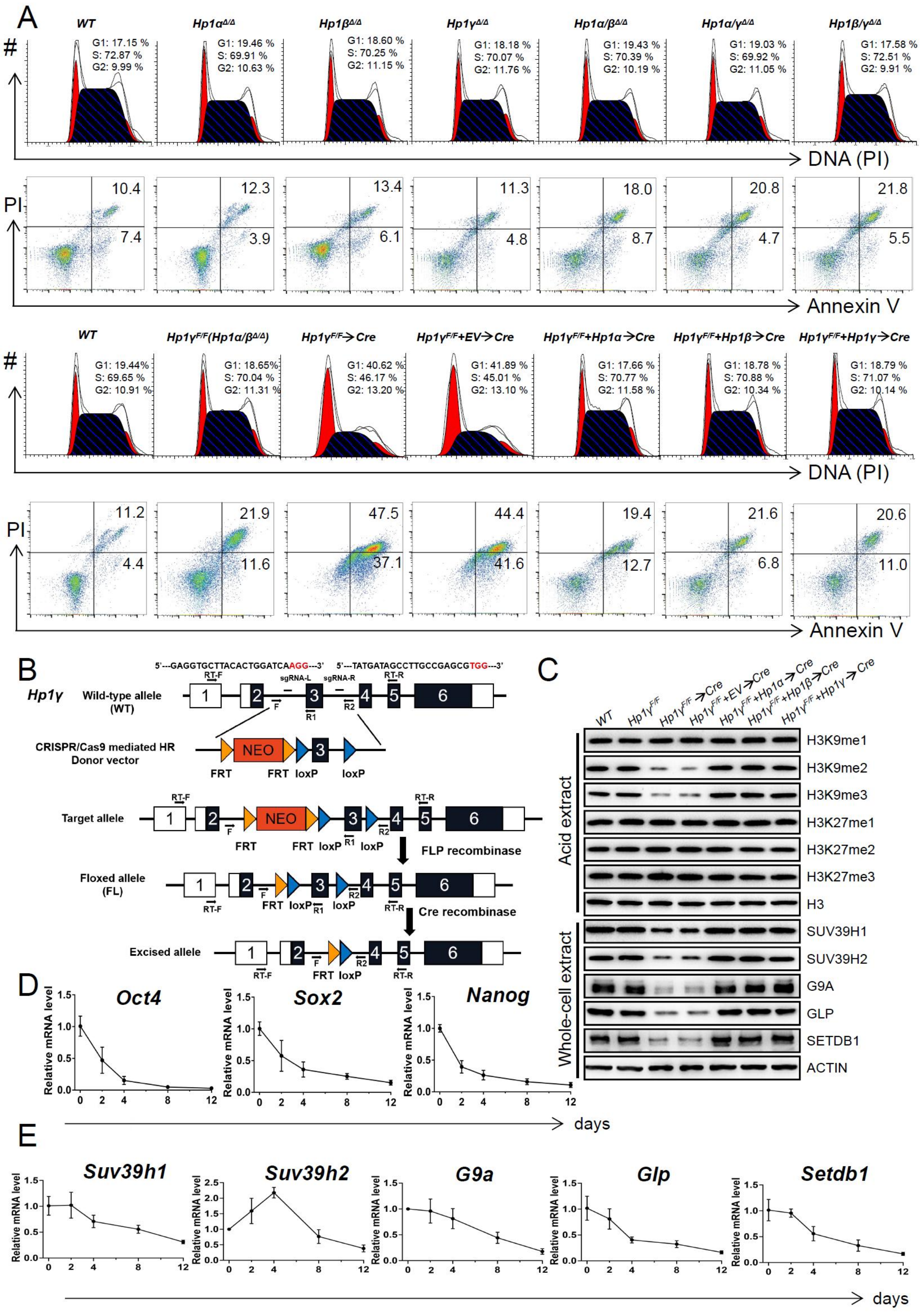
A
B
C
D
E
DNA (PI)
Annexin V
PI
Hp1γ
Wild-type allele (WT)
CRISPR/Cas9 mediated HR Donor vector
Target allele
Floxed allele (FL)
Excised allele
FLP recombinase
Cre recombinase
Acid extract
Whole-cell extract
H3K9me1
H3K9me2
H3K9me3
H3K27me1
H3K27me2
H3K27me3
H3
SUV39H1
SUV39H2
G9A
GLP
SETDB1
ACTIN
Oct4
Sox2
Nanog
Suv39h1
Suv39h2
G9a
Glp
Setdb1
Relative mRNA level
days

Figure S1. The HP1 family is required for maintenance of the pluripotent state in ESCs, related to Figure 1.

(A) Representative cell cycle profiles of propidium iodide–stained ESCs of indicated genotypes obtained by flow cytometric analysis (top). Flow cytometric analysis of apoptosis with annexin V and propidium iodide dual staining (bottom).

(B) Schematic illustration for generation of *Hp1γ* conditional allele via CRISPR/Cas9-mediated gene targeting. The sgRNA sequences were labeled in black and the PAM sequences in red.

(C) Western blot demonstrating the expression levels of selected histone modifications and H3K9 methyltransferases in cells of indicated genotypes. Actin and H3 were taken as loading controls.

(D) and (E) RT-qPCR analysis for (D) *Oct4*, *Sox2*, *Nanog* and (E) H3K9 methyltransferase transcripts in $Hp1\alpha/\beta^{\Delta/\Delta};Hp1\gamma^{F/F}$ ESCs after lenti-*Cre* infection. Relative expression is reflected as fold difference over uninfected ESCs normalized to *β-actin*. Data are expressed as means ± SEM of three biological replicates.

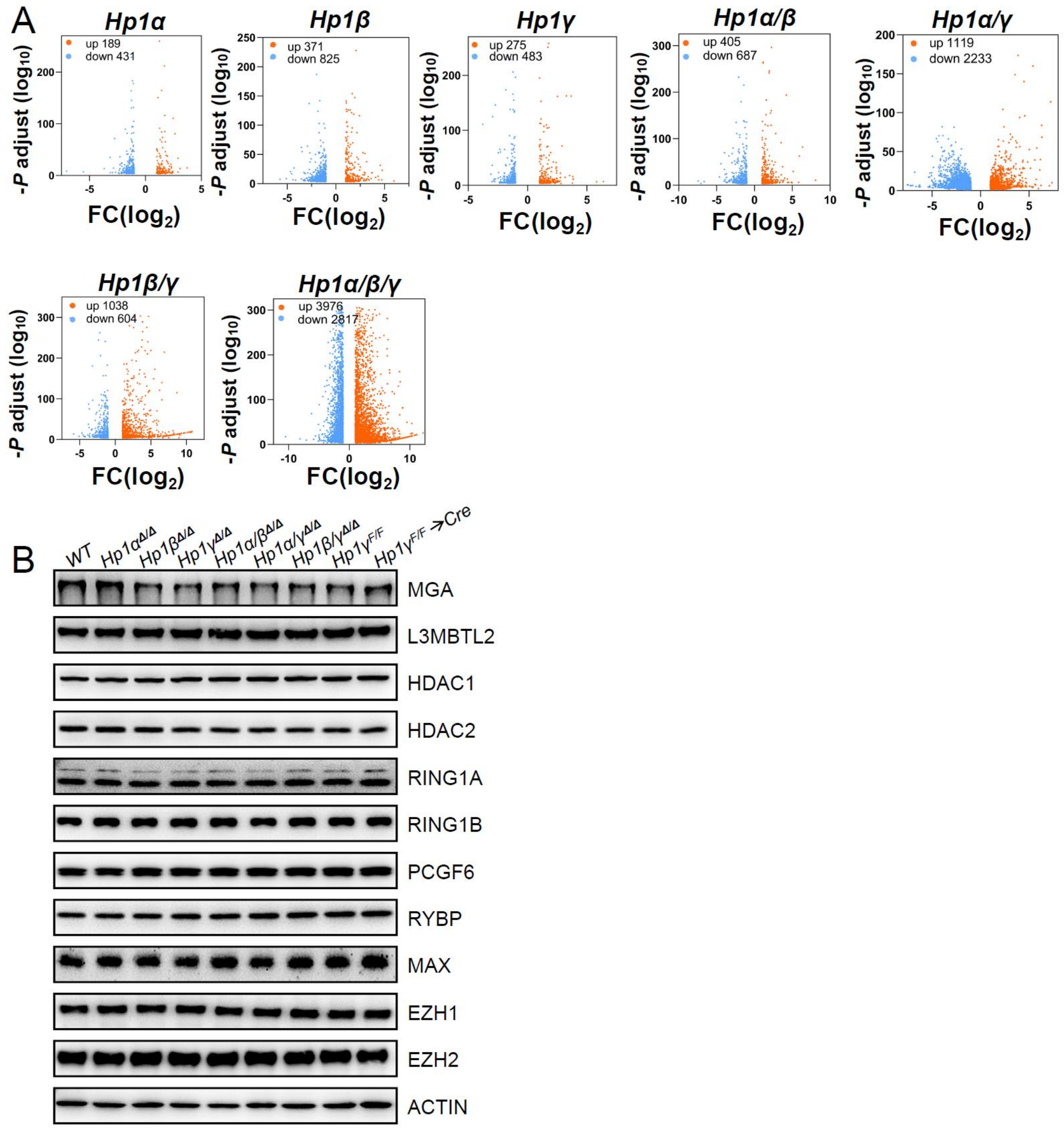

Figure S2. Complete loss of all three *Hp1* paralogs in ESCs exacerbates transcriptional changes in single or double mutants, related to Figure 2.

(A) Volcano plots showing the differentially expressed genes with 2-fold changes in ESCs of indicated genotypes. Blue and red points represent down-regulated represent up-regulated genes, respectively.

(B) Western blot analysis demonstrating expression levels of selected PRC1.6 and PRC2 members in ESCs of indicated genotypes. ACTIN was taken as a loading control.

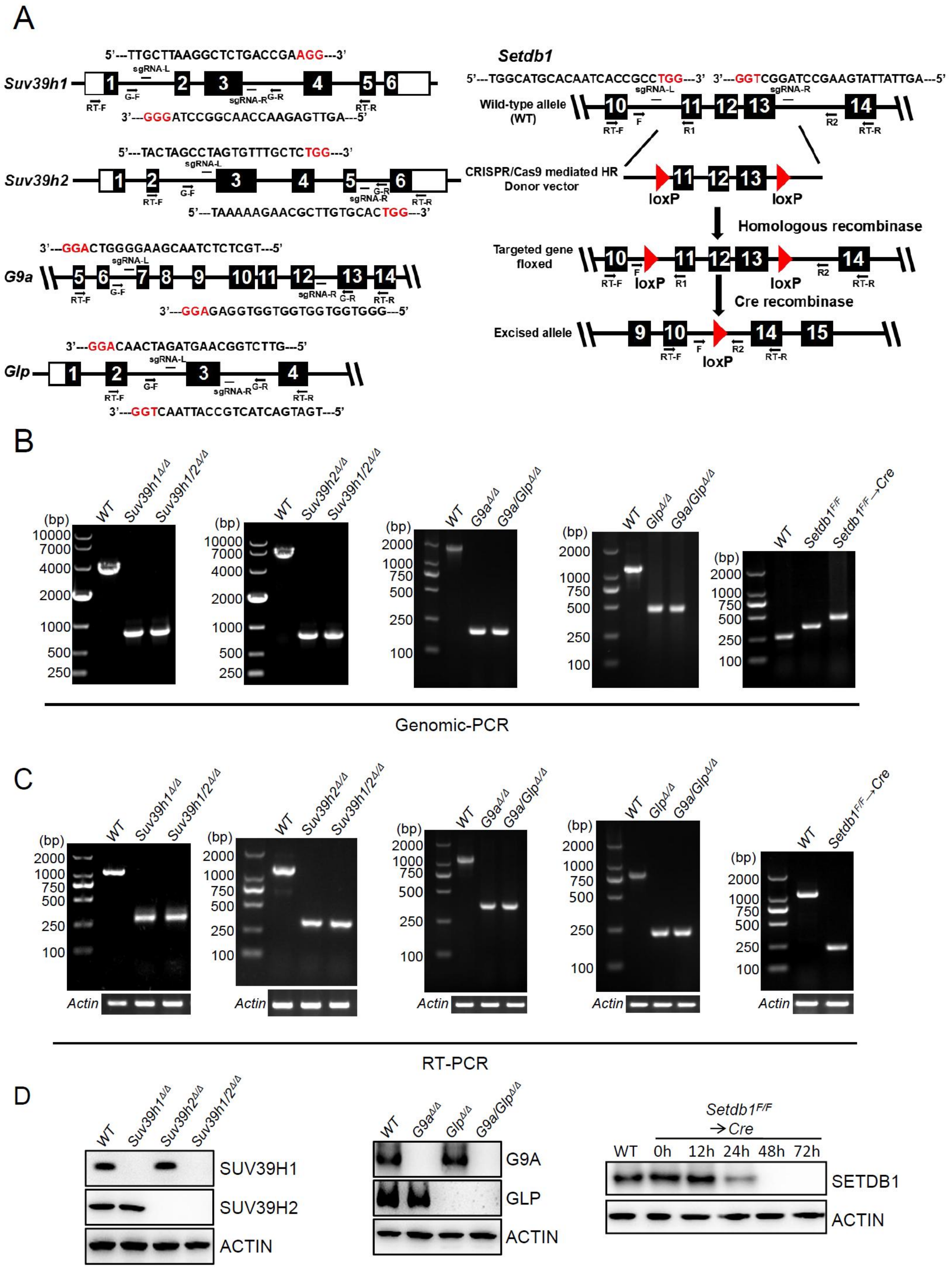

Figure S3. Generation of knockout ESC lines of *Suv39h1*, *Suv39h2*, *G9a*, *Glp* and *Setdb1*, related to Figure 5.

(A) Schematic representation of CRISPR/Cas9 mediated knockout approaches to generate ESCs deficient in *Suv39h1*, *Suv39h2*, *G9a, Glp* and *Setdb1*. SgRNA sequences are shown in black followed by the PAM sequences in red. The locations of genomic PCR primers (G-F, Forward; G-R, Reverse) are shown by arrows.

(B) Genotyping of ESCs with indicated gene deletions using primers located upstream

and downstream of the deleted region.

(C) RT-PCR analysis for residual mRNA revealed a shorter band in the mutants.

(D) Western blot showing the complete ablation of particular gene by using specific antibodiesas indicated. ACTIN was taken as a loading control.

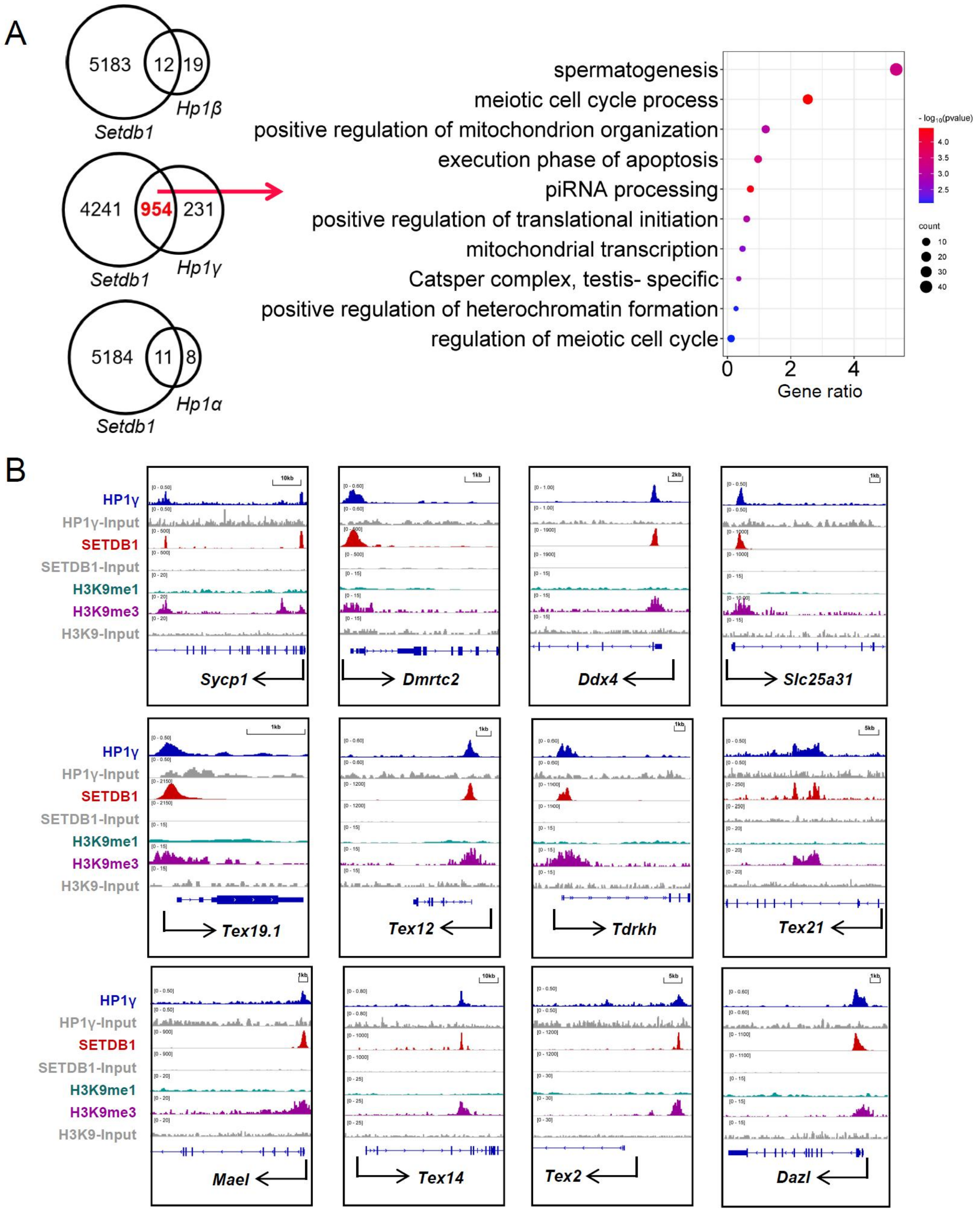

Figure S4. HP1γ and SETDB1 common targets are enriched for germ cell-specific genes in ESCs, related to Figure 6.

(A) Venn diagrams illustrating the overlap of HP1-bound genes and SETDB1-bound genes in ESCs (left). GO analysis of genes co-occupied by HP1γ and SETDB1 (right).

(B) Representative ChIP-seq tracks for HP1γ and SETDB1 at selected target gene loci in wild-type ESCs. Published ChIP-seq data for HP1 and SETDB1 were obtained from NCBI GEO database (accession number GSE94086, GSE71114, GSE108620, GSE126243, GSE54412 and GSE155062).

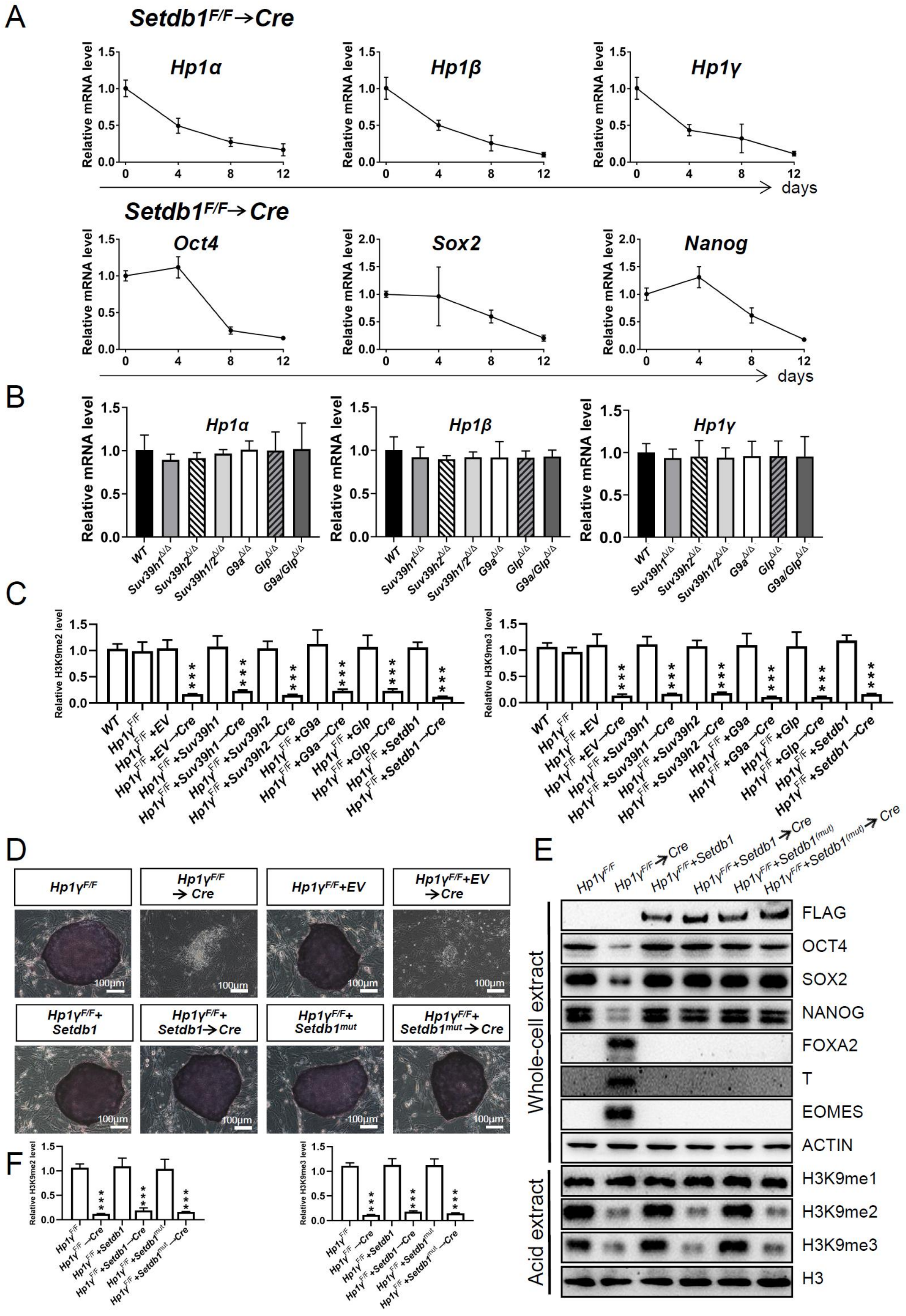

Figure S5. *Setdb1* deficiency results in the dynamic changes in mRNA expression of *Hp1* family genes, related to Figure 6.
(A and B) RT-qPCR analysis for *Hp1*, *Oct4*, *Sox2* and *Nanog* transcripts in *Setdb1*$^{F/F}$ ESCs after lenti-*Cre* infection (A) or in ESCs deficient for *Suv39h1*, *Suv39h2*, *G9a* and *Glp* (B). Relative expression is reflected as fold difference over uninfected or *wildtype* ESCs normalized to *β-actin*. Data are expressed as means ± SEM of three biological replicates. (C) Quantitation of the global levels of H3K9me2 and H3K9me3 in ESCs of indicated genotypes. (D) Representative AP staining images of ESC colonies of indicated genotypes on MEF feeders. Scale bar, 100 μm. *Hp1γ*$^{F/F}$: *Hp1α/β*$^{Δ/Δ}$*;Hp1γ*$^{F/F}$; *EV*: *empty vector*. (E) Western blot demonstrating the expression levels of ectopic *Setdb1*, pluripotency factors, germ layer markers, and histone modifications in cells of indicated genotypes. ACTIN and H3 were taken as the loading controls. *Hp1γ*$^{F/F}$: *Hp1α/β*$^{Δ/Δ}$*;Hp1γ*$^{F/F}$. (F) Quantitation of the levels of H3K9me2 and H3K9me3 in ESCs of indicated genotypes.

Table S1. Genes that are differentially expressed in *Hp1* single knockout vs. control ESCs.
Table S2. Genes that are differentially expressed in *Hp1* double knockout vs. control ESCs.
Table S3. Genes that are differentially expressed in *Hp1* triple knockout vs. control ESCs.
Table S4. Genes that are differentially expressed in *Setdb1*$^{Δ/Δ}$ vs. control ESCs.

Table S5: Key resources used in this study.

Supplemental Experimental Procedures

CRISPR/Cas9-mediated genomic editing

sgRNAs were designed by online CRISPR design tool (http://crispor.tefor.net/). A pair of 20bp-oligonucleotides was annealed and cloned into the BbsI-digested Cas9 and sgRNA-expressing PX459 vector (Addgene plasmid # 62988). sgRNA-expressing plasmids were verified by DNA sequencing. Targeting constructs with appropriate homology arms were generated by standard molecular cloning methods[1]. ESCs were transfected with 1 μg of each Cas9 guide, and 1.5 μg of targeting construct (where appropriate) using Lipofectamine 2000 (ThermoFisher) according to manufacturer's guidelines. The day after transfection, cells were subjected to puromycin (2 μg/ml) selection for 48 hours to eliminate any non-transfected cells. Approximately one week after seeding ESCs on feeder MEFs, individual clones were isolated, expanded, and PCR-screened for the desired genomic modification. All primers are listed in Table S5.

Cloning and plasmid generation

Full-length cDNAs encoding HP1, OCT4, SOX2, NANOG, SUV39H1, SUV39H2, G9A, GLP, and SETDB1 were RT–PCR amplified from mouse mRNA and cloned into lentivirus vector with FLAG epitope (DYKDDDDK) by standard DNA cloning methods. All plasmid

constructions were confirmed by DNA sequencing. Various truncations, deletion, and site mutations were constructed by PCR and confirmed by direct DNA sequencing. Lentiviral production and infection were performed as described previously[2].

Alkaline phosphatase staining

The ESCs were digested with trypsin and then seeded on mitomycin C-treated MEFs for 7-12 days to form colonies, which were washed with phosphate-buffered saline (PBS) twice and fixed with 4% paraformaldehyde for 2 min, and then incubated with staining solution following the protocol recommended by the manufacturer[1]. Finally, the stained colonies were photographed microscopically.

Growth curves

To perform growth curves, ESCs (5×10^4) were seeded in triplicate onto 10 cm MEF-coated dishes in 10 ml of ESC medium on day 0, with culture medium change every other day. Cells were harvested by trypsinization and counted (Trypan blue) every day for 12 days.

Flow cytometry

Cell cycle analysis

The cells were trypsinized, washed twice with ice-cold PBS, and fixed in 70% ethanol in PBS at -20 °C overnight. After fixation, cells were washed with ice-cold PBS and stained with propidium iodide (PI) staining solution, containing 50 μg/mL PI and 200 mg/mL RNase A, at 4°C for 30 min in the dark. Finally, cells were analyzed on a LSRFortessa flow cytometer equipped with Cell Quest software (BD Biosciences) as described previously [2].

Apoptosis analysis

Apoptosis analysis was performed by using Annexin V-FITC/PI apoptosis detection Kit (Yeasen 40302ES60). The cells to be analyzed were collected by trypsin digestion, washed twice with ice-cold PBS, and resuspended in 100 μl binding buffer at room temperature for 10 min in dark which contained 10 μl PI and 5 μl Annexin V-FITC. Fluorescence intensities were analyzed by LSRFortessa flow cytometry as described previously[2].

Western blot analysis

ESCs were lysed with RIPA buffer [50 mM Tris-HCl (pH 8.0), 1% Triton X-100, 0.1% SDS, 150 mM NaCl, 1% sodium deoxycholate, 1 mM EDTA, 10 mg/ml phenylmethylsulfonyl fluoride and Protease Inhibitor Mix (Sigma-Aldrich)] on ice for 30 min. After centrifugation at 13,000 rpm for 15 min at 4°C, the supernatant was collected. The samples were mixed with 5xloading buffer [2% SDS, 125 mM Tris-HCl (pH6.8), 10% glycerol, 1 mg/ml bromophenol blue and 2% β-mercaptoethanol], and heated at 95°C for 10 min. Then, an equal amount of protein (20–50 μg) was separated by with 5–15% SDS-PAGE, blotted to a PVDF membrane (Millipore) and blocked with 5% skim milk powder. The membranes were washed and incubated with the primary antibodies at 4°C overnight. The next day, immunoblotting was performed using the corresponding HRP-conjugated secondary antibody, followed by the visual detection of the protein bands using an enhanced

chemiluminescence detection kit. Antibodies used in this study are listed in Table S5.

Quantitative real-time PCR (RT-qPCR)

Total RNA was extracted by TRIzol reagent (Life Technologies) following the manufacturer's instructions. Complimentary DNA was reverse-transcribed from total RNA using HiScript II 1st Strand cDNA Synthesis Kit (Vazyme) according to the manufacturer's instructions. Samples were analyzed on an ABI 7500 Real Time PCR System (Applied Biosystems) using SYBR green PCR mastermix (Life Technologies) according to the manufacturer's protocol. Results were normalized to *Actin* mRNA levels. Primer sequences are available in Table S5.

RNA sequencing

The total RNA was extracted with TRIzol reagent (Invitrogen), and any contaminating DNA was digested with DNase I. Multiplexed Illumina sequencing libraries were prepared using the TruSeq Stranded Total RNA Library Prep Gold (Illumina, 20020598), and 150 bp paired-end reads were generated with Illumina HiSeq3000 sequencer with a depth of 50 million reads per sample. Differential expression analyses were performed using the R package DESeq2 v1.20 using default parameters. Genes with an absolute log2 fold change of 1 and FDR <0.05 were considered as significant difference. Three biological replicates of each sample were sequenced. The RNA-seq data were deposited at Gene Expression Omnibus under accession number GSE210606.

Analysis of publicly available ChIP-seq data

ChIP-Seq analysis of Publicly available datasets in ESCs were retrieved from the NCBI Gene Expression Omnibus (OCT4 and NANOG:GSE129721, HP1α:GSE94086, HP1β:GSE71114, HP1γ:GSE108620, H3K9me1:GSE54412, H3K9me3:GSE155062 and SETDB1:GSE126243). The ChIP-seq reads were all aligned to the mouse reference genome (GRCm38/mm10) via Bowtie (v2.3.5) in the single-end mode. Then the reads with low quality (MAPQ <20) were removed with the Bam/Sam filter software (version: 1.6). To better visualize the results, alignment files in the BAM format were converted to the read coverage files (BIGWIG format) by the bamCoverage in DeepTools. Subsequently， the converted BIGWIG files were visualized with Integrative Genome Viewer (IGV). And data ranges were set for the appropriate values to underscore differences between each factor level at the target locus. The MACS-determined peak positions were downloaded from ChIP-Atlas (http://chip-atlas.org). Peak overlaps were analyzed using VennDiagram program of Origin (version 2021).

Embryoid body formation

To induce the EB formation, modified hanging drop method from a previously described protocol was followed[2]. Trypsinized cells were suspended in differentiation medium (50 μg/ml Ascorbic acid, 15% fetal bovine serum, 200 μg/ml iron saturated holo-transferrin, 2 mM L-Glutamine, 450 μM monosulfide glycerol, 100 U/ml penicillin-streptomycin, non-essential amino acids and 0.1 mM β-mercaptoethanol) to a concentration of 20,000 cells/ml. 30 μl drops (600 cells) were then placed on the lid of a non-adherent Petri

dish and the lid was upside down. After 72 h incubation, EBs formed at the bottom of the drops were collected and cultured on the rotating shaker at the speed of 60 rpm, with the medium being changed every other day. On day 7 and 12, EBs were collected for morphological imaging and total RNA extraction (Trizol). The genes of interest were analyzed by RT-qPCR. All primers are listed in Table S5.

Teratoma assay

Teratoma formation assay was performed as described previously [1,2]. Briefly, ESCs were MEF depleted and 1×10^6 cells were injected subcutaneously into 6-week-old nude mice. After 4 weeks, the teratomas were dissected and fixed in 4% paraformaldehyde for 6 hours at 4℃. Paraffin-embedded tissue was sliced into sections with 5 μm thickness and stained with hematoxylin and eosin.

Supplementary References

1. Qin, J., Wang, C., Zhu, Y., Su, T., Dong, L., Huang, Y., and Hao, K. (2021). Mga safeguards embryonic stem cells from acquiring extraembryonic endoderm fates. Sci Adv *7*. 10.1126/sciadv.abe5689.
2. Qin, J., Whyte, W.A., Anderssen, E., Apostolou, E., Chen, H.H., Akbarian, S., Bronson, R.T., Hochedlinger, K., Ramaswamy, S., Young, R.A., and Hock, H. (2012). The polycomb group protein L3mbtl2 assembles an atypical PRC1-family complex that is essential in pluripotent stem cells and early development. Cell Stem Cell *11*, 319-332. 10.1016/j.stem.2012.06.002.
